# Periodontitis aggravates pulmonary fibrosis by *Porphyromonas gingivalis*-promoted infiltration of neutrophils and Th17 cells

**DOI:** 10.3389/fcimb.2025.1595500

**Published:** 2025-05-27

**Authors:** Hui-Lin Ye, Xiao-Qian Meng, Huxiao Li, Xiaoli Sun, Wen-Zhen Lin, Lu-Jun Zhou, Jun Zhang, Chenchen Hou, Shuo Xu, Bo-Yan Chen, Che Qiu, Yu-Lin Li, Yong-Li Wang, Li-Feng Yan, Sheng-Zhong Duan

**Affiliations:** ^1^ Department of Endodontics, Shanghai Ninth People’s Hospital, College of Stomatology, Shanghai Jiao Tong University School of Medicine, Shanghai, China; ^2^ Laboratory of Oral Microbiota and Systemic Diseases, Shanghai Ninth People’s Hospital, College of Stomatology, Shanghai Jiao Tong University School of Medicine, National Center for Stomatology, National Clinical Research Center for Oral Diseases; Shanghai Key Laboratory of Stomatology, Shanghai, China; ^3^ Department of Periodontology, Shanghai Ninth People’s Hospital, Shanghai Jiao Tong University School of Medicine, Shanghai, China; ^4^ Department of Respiratory and Critical Care Medicine, Shanghai Pulmonary Hospital, Tongji University School of Medicine, Shanghai, China; ^5^ Department of Respiratory and Critical Care Medicine, Shanghai Ninth People’s Hospital, Shanghai Jiao Tong University School of Medicine, Shanghai, China; ^6^ Stomatology Hospital, School of Stomatology, Zhejiang University School of Medicine, Zhejiang Provincial Clinical Research Center for Oral Diseases, Key Laboratory of Oral Biomedical Research of Zhejiang Province, Cancer Center of Zhejiang University, Engineering Research Center of Oral Biomaterials and Devices of Zhejiang Province, Hangzhou, China

**Keywords:** periodontitis, idiopathic pulmonary fibrosis, *Porphyromonas gingivalis*, neutrophils, Th17 cells

## Abstract

**Introduction:**

Idiopathic pulmonary fibrosis (IPF) is a fatal interstitial lung disease. However, the pathogeny of IPF is poorly understood, and therapeutic options are very limited. Periodontitis (PD) is a chronic inflammatory disease that leads to dysbiosis of both the oral microbiome and host immune responses. While previous studies have suggested a PD-IPF association, insights into the mechanisms remain limited.

**Methods:**

The PD mouse model was established by the ligation of molars and oral inoculation of subgingival plaques from PD patients and subsequently incorporated with a bleomycin-induced pulmonary fibrosis model. The effect of PD on pulmonary fibrosis was determined. Changes of immune cells were analysed using flow cytometry. Moreover, the microbiome changes of the lungs and oral cavity were assessed by 16S rRNA gene sequencing and fluorescence in situ hybridization. Finally, the effect and mechanism of the specific PD pathogen on pulmonary fibrosis were determined.

**Results:**

PD significantly aggravated pulmonary fibrosis in mice by increasing the infiltration of neutrophils and Th17 cells. Neutrophils and Th17 cells are critical in PD-induced aggravation of pulmonary fibrosis, and Th17 cells regulate neutrophils via IL-17A. The PD pathogen *Porphyromonas gingivalis (Pg)* was detected enriched in both the oral cavity and lungs. *Pg* was further determined to exacerbate pulmonary fibrosis by increasing the expansion of neutrophils and Th17 cells in mice.

**Conclusion:**

PD aggravates pulmonary fibrosis in mice, which is likely induced by *Pg*-promoted infiltration of neutrophils and Th17 cells. Treatment targeting PD or *Pg* might be a promising strategy to clinically ameliorate IPF.

## Introduction

Idiopathic pulmonary fibrosis (IPF) is the most common and morbid type of idiopathic interstitial pneumonia (Podolanczuk et al., 2023). IPF is a chronic, progressive and incurable pulmonary disease. The prognosis of IPF patients is poor. Without treatment, the median survival of IPF patients is merely 3–5 years, which is even worse than the prognosis of many cancers (Bonella et al., 2023). The etiology of IPF is very complicated and poorly understood, and risk factors include genetic mutation, environmental exposures, dysbiosis of lung microbiome, and inflammation (Moss et al., 2022; Karampitsakos et al., 2023). Consequently, current treatments for IPF are limited to pirfenidone and nintedanib, which to some extent slow the development of IPF but never improve or stabilize pulmonary function (Spagnolo et al., 2021). Therefore, identifying the pathogenesis of IPF and further developing promising treatments for IPF are urgently needed.

The respiratory system is adjacent to oral cavity and has a close relationship with it. Oral diseases can disrupt the homeostasis of the lungs and even aggravate various lung diseases. Periodontitis (PD) is one of the most common oral diseases and is a complex inflammatory disease (Meyle and Chapple, 2015). PD causes the loss of teeth and periodontal tissues, not only causing oral dysfunction but also endangering overall health as a chronic lesion (Hajishengallis and Chavakis, 2021). PD is accompanied by dysbiosis of both the oral microbiome and host immune responses, which causes systemic dissemination of oral pathogens and related virulence factors as well as immune cells and inflammatory cytokines (Kinane et al., 2017). Consequently, invasion of these oral pathogens and associated inflammatory cascades can promote the development of systemic diseases (Teles et al., 2022). In recent decades, various studies have indicated correlations between periodontitis and at least 43 systemic diseases, such as diabetes, inflammatory bowel disease, hypertension, and respiratory diseases (Slots, 2017).

Recently, studies have indicated a potential correlation between PD and IPF. The previous clinical study revealed that 32.84% of lung microbiome genes are derived from the oral microbiome, and compared with the smoking group (control group), 38% of the differentially enriched genes in the IPF group are from the oral microbiome (Tong et al., 2019). Other studies have illustrated that *Streptococcus* and *Veillonella* are significantly enriched in IPF patients compared with control subjects (Molyneaux et al., 2014; Invernizzi et al., 2021). *Streptococcus* and *Veillonella* are common oral bacteria and are closely associated with PD (Kumar et al., 2005; Koyanagi et al., 2013). Adult cystic fibrosis patients often have poor oral hygiene habits, more tooth plaques and calculus (Coffey et al., 2024), and exhibit widespread presence of periodontal pathogens in subgingival biofilms and in sputum (Rivas Caldas et al., 2015; Pawlaczyk-Kamieńska et al., 2019). Due to anatomic proximity, the oral microbiota may enter the lungs by microaspiration, and the lung microbiota resembles the oral microbiota more than the nasal microbiota does (Bassis et al., 2015; Gaeckle et al., 2020). Clinical and animal studies have demonstrated that incremental lung bacteria are strongly related to faster development and a greater rate of IPF mortality (O’Dwyer et al., 2019; Amati et al., 2022). PD pathogens such as *Porphyromonas gingivalis* (*Pg*) and *Fusobacterium nucleatum* have been verified to be present in the lungs and to promote lung diseases by increasing inflammation (Li et al., 2021; Feng et al., 2024). However, there is no direct evidence indicating whether PD is correlated with IPF and whether PD exacerbates IPF.

In this study, we aimed to reveal the relationship between PD and IPF and identify the underlying mechanisms. First, we investigated the effects of PD on BLM-induced (bleomycin-induced) pulmonary fibrosis in mice. Next, we analyzed changes of immune cells, and determined critical immune cells and their interactions in the PD-induced aggravation of pulmonary fibrosis. Finally, we identified a key PD pathogen enriched in both the oral and lungs and verified its importance and mechanisms for promoting pulmonary fibrosis in mice.

## Materials and methods

### Mice and treatments

Eight-week-old C57BL/6 male mice were ordered from Beijing Vital River Laboratories (Beijing, China). The mice were housed in a specific pathogen-free (SPF) facility. Mice aged 10–12 weeks were used in the study. PD was established in the mice as previously described (Bai et al., 2022). The bilateral maxillary second molars of the mice were ligatured with 5–0 silk. Then, mice were orally inoculated with subgingival plaques (1x10^9^ colony-forming units per mouse) from PD patients every other day for 2 weeks. Patients with moderate to severe PD were recruited and those who smoke, suffer systemic diseases, have less than 8 teeth, take antibiotics or probiotics and undergo periodontal treatments within the last 6 months were excluded. Subgingival plaques were then collected from PD patients and mixed well and stored in the -80°C freezer in advance. Subsequently, pulmonary fibrosis was induced in the mice with BLM (HY-17565A, MedChemExpress, Shanghai, China). Briefly, mice were first anaesthetized with isoflurane gas (4%) and then intratracheally administered BLM (0.5 mg/kg body weight, 30 μl per mouse) or saline control by an atomizing needle (Bio Jane Trading Co., Ltd., Shanghai, China). Mice were sacrificed 3 weeks later or at the indicated time points for further analysis.

The *Pg*-induced PD mouse model was established by ligation of bilateral maxillary second molars with 5–0 silk sutures as previously described and then orally inoculated with *Pg* (ATCC 33277, 1x10^9^ colony-forming units per mouse) every other day for 2 weeks. Then BLM was intratracheally administered, and the sutures were maintained until the end of the experiment. The Institute of Animal Care and Use Committee (IACUC) of Cyagen Biosciences, China (Approval No. ICU21-0080), approved the conduct of all the animal experiments. Collecting subgingival plaques from PD patients was approved by the Institutional Review and Ethics Board of Shanghai Ninth People’s Hospital, Shanghai Jiao Tong University School of Medicine (No. SH9H-2021-T115–1). All participants provided informed consent.

To deplete neutrophils, mice were intraperitoneally injected with 200 µg of anti-mouse Ly6G antibody (BE0075-1, BioXCell) every 2 days since BLM administration to the end of the experiment. To neutralize IL-17A, mice were intraperitoneally injected with 200 µg of anti-mouse IL-17A antibody (BE0173, BioXCell) every 3 days since BLM administration until the end of the experiment.

### Analysis of alveolar bone loss

To determine PD-induced alveolar bone loss as previously described (Abe and Hajishengallis, 2013), maxillae of mice were acquired, and the gingiva were removed, followed by bleaching and then rinsing. 1% methylene blue was then used to stain the maxillae. The staining maxillae were captured by a Leica digital camera (Leica, Germany). The distance between the cementoenamel junction (CEJ) and alveolar bone crest (ABC) was measured by ImageJ software (National Institutes of Health, Bethesda, USA).

### Histology and hydroxyproline quantification

Lung lobes were fixed by 4% paraformaldehyde, dehydrated and then embedded in paraffin. Paraffin-embedded sections were subsequently performed with H&E staining or Masson’s trichrome staining. All images were obtained with a Leica DMi8 microscope (Leica, Wetzlar, Germany). The level of interstitial fibrosis was assessed as acknowledged protocols (Hübner et al., 2008). The hydroxyproline level was quantified by a hydroxyproline assay kit (A030-3-1, Nanjing Jiancheng, China).

### Quantitative real−time PCR

RNA of lung tissues was isolated by TRIzol reagent (15596018, Invitrogen, USA). RNA was then reverse-transcribed to cDNA by a reverse transcription kit (Takara, Shiga, Japan). A LightCycler 480 II (Roche) was used to perform real-time PCR. Primers used in this study were listed in **Supplementary Table S1**.

### Western blot analysis

Lung tissues were homogenized using RIPA lysis buffer containing phenylmethylsulfonylfluoride (PMSF, Sigma–Aldrich) and protease inhibitor (Med Chem Express). Protein concentration was then measured by the BCA protein assay kit (88512, Thermo Fisher Scientific). The 10% SDS–PAGE was used to separate proteins. Detection was performed with ECL Western Blotting Substrates (Thermo Fisher Scientific). The antibodies used in this study were anti-TGF-β1 (ab215715, Abcam), anti-Fibronectin (ab2413, Abcam) and anti-GAPDH (14c10, Cell Signaling Technology).

### Preparation of single-cell suspensions from tissues

Single-cell suspensions from the lungs were prepared according to previous protocol (Jungblut et al., 2009). The lungs were put in a gentleMACS C Tube (130-093-237, Miltenyi Biotec) containing 5 mL of HEPES buffer supplemented with DNase I (80 U/mL, A37780050, applichem) and collagenase IV (300 U/ml, A004186-0001, worthington). The C Tube was attached to the gentleMACS Dissociator (130-093-235, Miltenyi Biotec) to run the program “m_lung_01”. Then the lung tissues were digested for 30 minutes with agitation at 37°C and followingly run the program “m_lung_02” to obtain suspensions. PBS was added to terminate the digestion, and the suspensions were filtered by a cell strainer to acquire single lung cells. The cervical lymph nodes (cLNs) of the mice were ground on a cell strainer and rinsed with PBS to obtain single cells. Bone marrow cells were prepared by flushing the femur with a syringe.

### Flow cytometry analysis

Single-cell suspensions from the lungs, cLNs and bone marrow were centrifuged and stained with Live/Dead Fixable Viability Stain 510 (564406, BD Biosciences). The cell samples were incubated with CD16/32 antibodies (101320, Biolegend) to block Fc receptors before surface staining and then stained with antibodies. For cytokine staining, cells were stimulated with the cell activation cocktail (423303, BioLegend), washed with PBS and finally stained with antibodies. Antibodies against the following molecules were used in this study: CD45 (557659, Biosciences), CD11b (101205, Biolegend), Ly6G (127616, Biolegend), Ly6C (128018, Biolegend), CD64 (139321, Biolegend), CD11c (117333, Biolegend), I-A/I-E (562564, Biosciences), SiglecF (155506, Biolegend), CD3 (100203, Biolegend), CD4 (100540, Biolegend), CD8 (100722, Biolegend), B220 (103251, Biolegend), F4/80 (123116, Biolegend), CD115 (135515, Biolegend), IFN-γ (505810, Biolegend) and IL-17A (506903, Biolegend). Samples were run on a BD LSRFortessa X-20 flow cytometer (BD Biosciences).

### Analysis of microbiome

Total genomic DNA from lungs and oral silk ligatures was extracted by an OMEGA Soil DNA Kit (M5635-02, Omega Bio-Tek). Qualified DNA samples were performed PCR amplification of the nearly full-length bacterial 16S rRNA genes. PCR amplicons were then sequenced by the PacBio Sequel platform (Shanghai Personal Biotechnology Co., Ltd., China). Microbiome bioinformatics was performed with QIIME software and R packages (v3.2.0). The alpha diversity was analysed by the ASV table in QIIME2, and beta diversity was performed with Bray–Curtis metrics. Differentially abundant taxa between groups were analysed by linear discriminant analysis effect size (LEfSe) with the default parameters. The raw sequences were deposited in the NCBI Sequence Read Archive database with the accession number PRJNA1138471.

### Fluorescence *in situ* hybridization

Identifying the presence of *Pg* was by fluorescence *in situ* hybridization (FISH) according to a published protocol (Rudney et al., 2005; Niu et al., 2024). Lung tissue slides were incubated with a specific Alexa Fluor 594-labelled *Pg* probe (5’-CAATACTCGTATCGCCCGTTATTC-3’) at 46°C overnight. Samples were washed with solution (100 mM Tris–HCl, 0.9 M NaCl, pH 7.5) for 25 min at 48°C, then distilled water, air-dried and counterstained with DAPI (Invitrogen). Images were obtained with a Leica DMi8 microscope (Leica, Wetzlar, Germany).

### Statistical analysis

The experimental data are presented as the mean ± SEM. Unpaired Student’s t test was used for two-group comparisons. One-way ANOVA was used for comparisons among more than two groups. All statistical analyses were performed with GraphPad Prism software. Values of P < 0.05 were considered to indicate statistical significance. Information about the statistical details and methods is also included in Figure legends.

## Results

### PD aggravates BLM-induced pulmonary fibrosis

**Figure 1:**
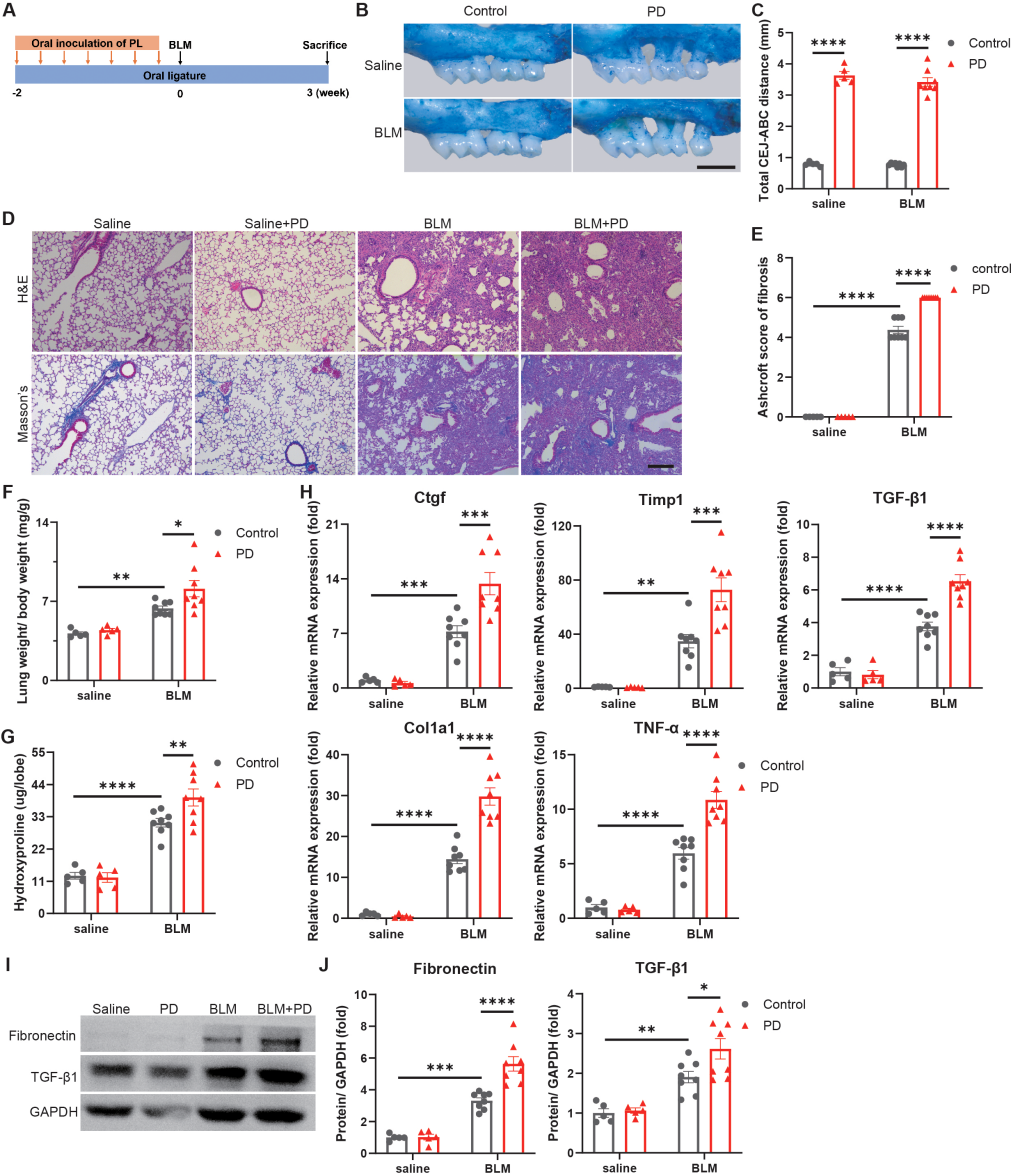
Periodontitis (PD) aggravates bleomycin-induced pulmonary fibrosis in mice. **(A)** Schematic illustration of the experimental design. PD was induced in mice by silk ligatures tied around molars and oral inoculation of subgingival plaques (PL) from patients with PD. Pulmonary fibrosis was induced by intratracheally administration of bleomycin (BLM). **(B)** Representative methylene blue staining of alveolar bones. Scale bar: 1 mm. **(C)** Quantification of the distance from the buccal cementoenamel junction (CEJ) to alveolar bone crest (ABC). **(D)** Representative images of H&E and Masson’s trichrome staining of mouse lung sections. Scale bar: 200 µm. **(E)** Quantitative analysis of pulmonary fibrosis as Ashcroft score based on Masson’s trichrome staining. **(F)** Lung weight to body weight ratio of mice. **(G)** Quantification of hydroxyproline content in mouse lungs. **(H)** qRT–PCR analysis of fibrotic genes in mouse lungs. **(I–J)** Representative Western blotting analysis **(I)** and quantifications **(J)** of Fibronectin and TGF-β1 in mouse lungs. n=5:5:8:8. Data are presented as mean ± SEM. One-way ANOVA was used for statistical analysis. *P < 0.05, **P < 0.01, ***P < 0.001, and ****P < 0.0001.

To determine the impact of PD on pulmonary fibrosis, the PD mouse model combined with pulmonary fibrosis was established. The molars of the mice were ligatured with silk sutures, and subgingival plaques from PD patients were inoculated in mouse oral cavity to establish PD with humanized oral microbiota, followed by BLM administration to induce pulmonary fibrosis (**Figure 1A**). PD caused severe alveolar bone loss (**Figure 1B**) and significantly increased the distance between the CEJ and ABC in mice (**Figure 1C**).

Moreover, the results of H&E and Masson’s trichrome staining indicated that PD induced more severe destruction of lung architecture and obviously increased collagen accumulation in mouse lungs (**Figure 1D**). Ashcroft scores, which assess the severity of fibrosis, also indicated that PD aggravated pulmonary fibrosis (**Figure 1E**). The lungs in the BLM combined with PD group were obviously heavier than those in the BLM group (**Figure 1F**). The hydroxyproline content, a marker of lung fibrosis severity, was markedly greater in lungs of the BLM combined with PD group than the BLM group (**Figure 1G**). Consistent with these observations, PD also obviously promoted the expression of fibrotic genes (**Figure 1H**). The western blot results also demonstrated that PD significantly increased the fibrotic protein levels such as Fibronectin and TGF-β1 (**Figures 1I, J**). Collectively, these results illustrated that PD exacerbated pulmonary fibrosis in mice.

### PD increases the infiltration of neutrophils and Th17 cells in mice with pulmonary fibrosis

**Figure 2:**
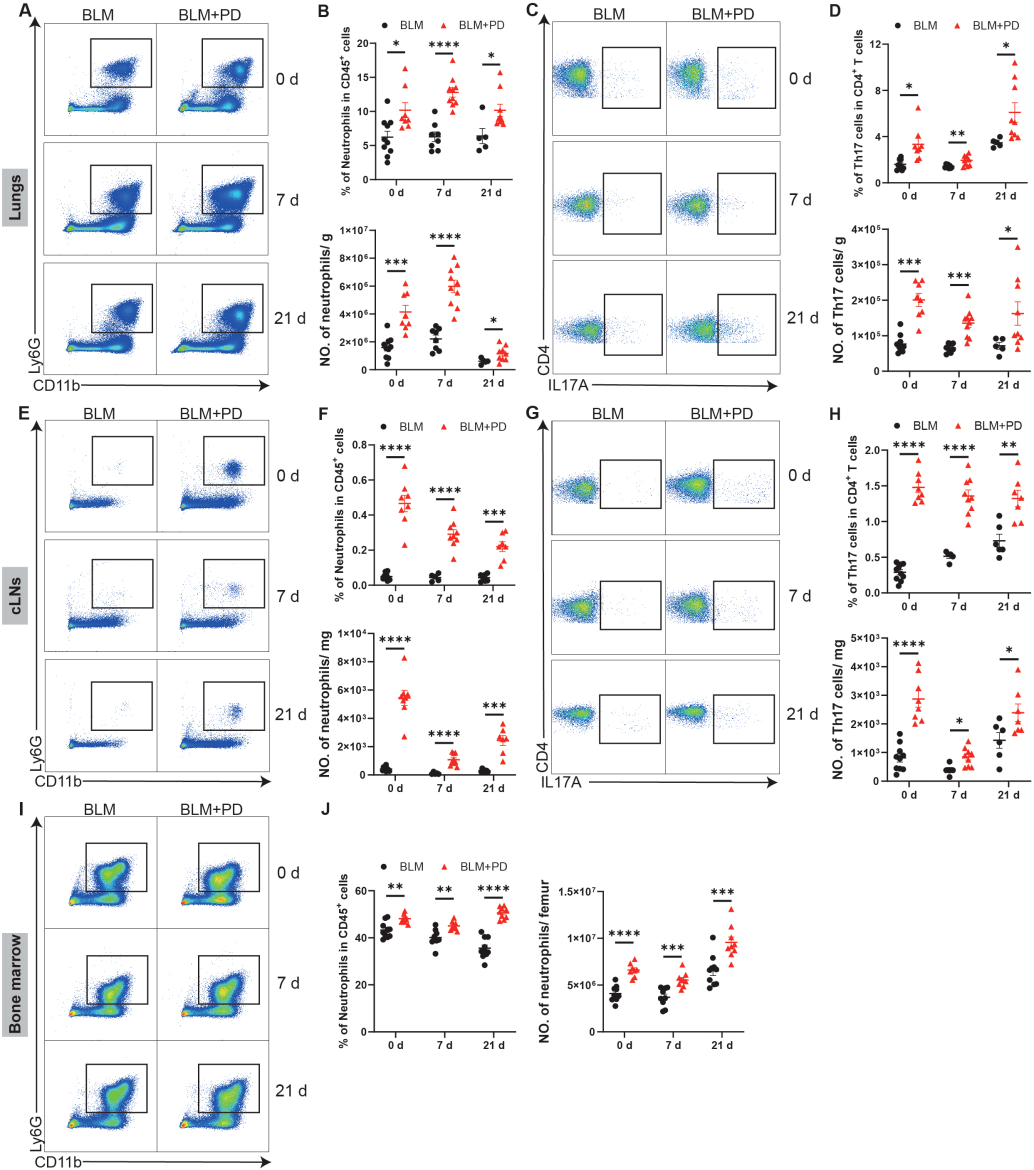
PD increases the infiltration of neutrophils and Th17 cells in mice with pulmonary fibrosis. **(A)** Representative flow cytometry plots of neutrophils in mouse lungs at 0 day, 7 days and 21 days after BLM administration. **(B)** Quantifications of neutrophils in mouse lungs as percentages and numbers. NO., number. **(C)** Representative flow cytometry plots of Th17 cells in mouse lungs at 0 day, 7 days and 21 days after BLM administration. **(D)** Quantifications of Th17 cells in mouse lungs as percentages and numbers. **(E)** Representative flow cytometry plots of neutrophils in cervical lymph nodes (cLNs) at 0 day, 7 days and 21 days after BLM administration. **(F)** Quantifications of neutrophils in mouse cLNs as percentages and numbers. **(G)** Representative flow cytometry plots of Th17 cells in mouse cLNs at 0 day, 7 days and 21 days after BLM administration. **(H)** Quantifications of Th17 cells in mouse cLNs as percentages and numbers. **(I)** Representative flow cytometry plots of neutrophils in mouse bone marrow at 0 day, 7 days and 21 days after BLM administration. **(J)** Quantifications of neutrophils in mouse bone marrow as percentages and numbers. n=5-10. Data are presented as mean ± SEM. Student’s t-test was used for statistical analysis. *P < 0.05, **P < 0.01, ***P < 0.001, and ****P < 0.0001.

Chronic inflammation is generally considered as a risk factor of pulmonary fibrosis, and many studies have verified that various immune cells are important for lung fibrosis (Yang et al., 2019). To explore immune mechanisms underlying PD-induced aggravation of pulmonary fibrosis, we next analyzed changes of immune cells. Myeloid cells and lymphoid cells in the lungs were analysed in an unbiased manner via flow cytometry at 0, 7 and 21 days after BLM treatment. Both the percentage and number of neutrophils were markedly greater in the lungs of the BLM+PD group than the BLM group at 0, 7 and 21 days after BLM administration (**Figures 2A, B**). Compared with the BLM group, the percentage and number of Th17 cells were also significantly greater in the BLM+PD group at the 3 indicated time points (**Figures 2C, D**). However, the percentage of other myeloid cells and lymphoid cells in the lungs did not always show the same significant trend as the number at the 3 indicated time points (**Supplementary Figure S1**). This indicated that neutrophils and Th17 cells were the most important immune cells that mediated the PD-induced aggravation of pulmonary fibrosis.

Additionally, myeloid cells and lymphoid cells in the cLNs and bone marrow of mice were also analysed at 0, 7 and 21 days after BLM administration. PD markedly increased neutrophils and Th17 cells in the cLNs (**Figures 2E–H**). PD also led to obvious changes in other myeloid cells and lymphoid cells in the cLNs (**Supplementary Figures S2A–L**). A significant increase in B cells was also detected among the changes caused by PD in the cLNs (**Supplementary Figures S2A–F**). PD also affected myeloid cells and lymphoid cells in the bone marrow of mice at different time points (**Supplementary Figures S2M–R**). Compared with the BLM group, the percentage and number of neutrophils in the bone marrow were markedly greater in the BLM+PD group (**Figures 2I, J**). Together, these results demonstrated that for mice with pulmonary fibrosis, PD increased the expansion of neutrophils and Th17 cells in lungs, and that cLNs and bone marrow were likely the sources of increased neutrophils and Th17 cells.

### Neutrophils and Th17 cells are critical to PD-induced aggravation of pulmonary fibrosis, and Th17 cells regulate neutrophils via IL-17A

**Figure 3:**
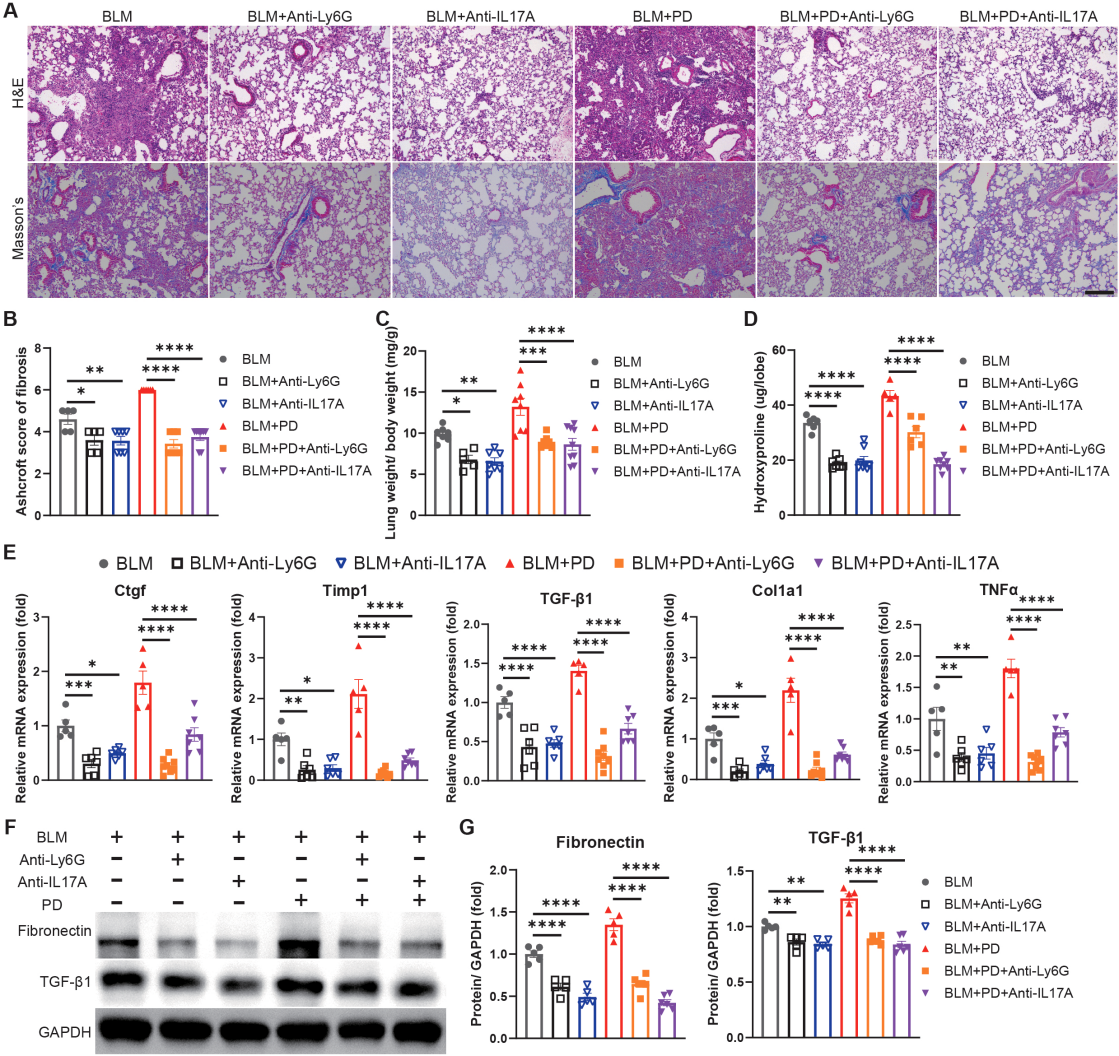
Neutrophils and Th17 cells mediate PD-induced aggravation of pulmonary fibrosis. **(A)** Representative images of H&E and Masson’s trichrome staining of mouse lung sections. Anti-Ly6G, anti-Ly6G antibody; Anti-IL17A, anti-IL17A antibody. Scale bar: 200 µm. **(B)** Quantitative analysis of pulmonary fibrosis as Ashcroft score based on Masson’s trichrome staining. **(C)** Lung weight to body weight ratio of mice. **(D)** Quantification of hydroxyproline content in mouse lungs. **(E)** qRT–PCR analysis of fibrotic genes in mouse lungs. **(F-G)** Representative Western blotting analysis **(F)** and quantifications **(G)** of Fibronectin and TGF-β1 in mouse lungs. n=5-9. Data are presented as mean ± SEM. One-way ANOVA was used for statistical analysis. *P < 0.05, **P < 0.01, ***P < 0.001, and ****P < 0.0001.

To further investigate the importance of neutrophils and Th17 cells in PD-induced aggravation of pulmonary fibrosis, we depleted neutrophils or IL-17A (mainly generated by Th17 cells) to assess the severity of lung fibrosis in mice. The lung damage and collagen accumulation were notably attenuated in the BLM+PD+Anti-Ly6G group and BLM+PD+Anti-IL17A group than those in the BLM+PD group (**Figures 3A, B**). The weights of the lungs and the hydroxyproline level were significantly reduced in PD mice after depletion of neutrophils or IL-17A (**Figures 3C, D**). Consistent with above results, the expression of fibrosis-associated genes and proteins also markedly reduced in PD mice after depletion of neutrophils or IL-17A (**Figures 3E–G**).

**Figure 4:**
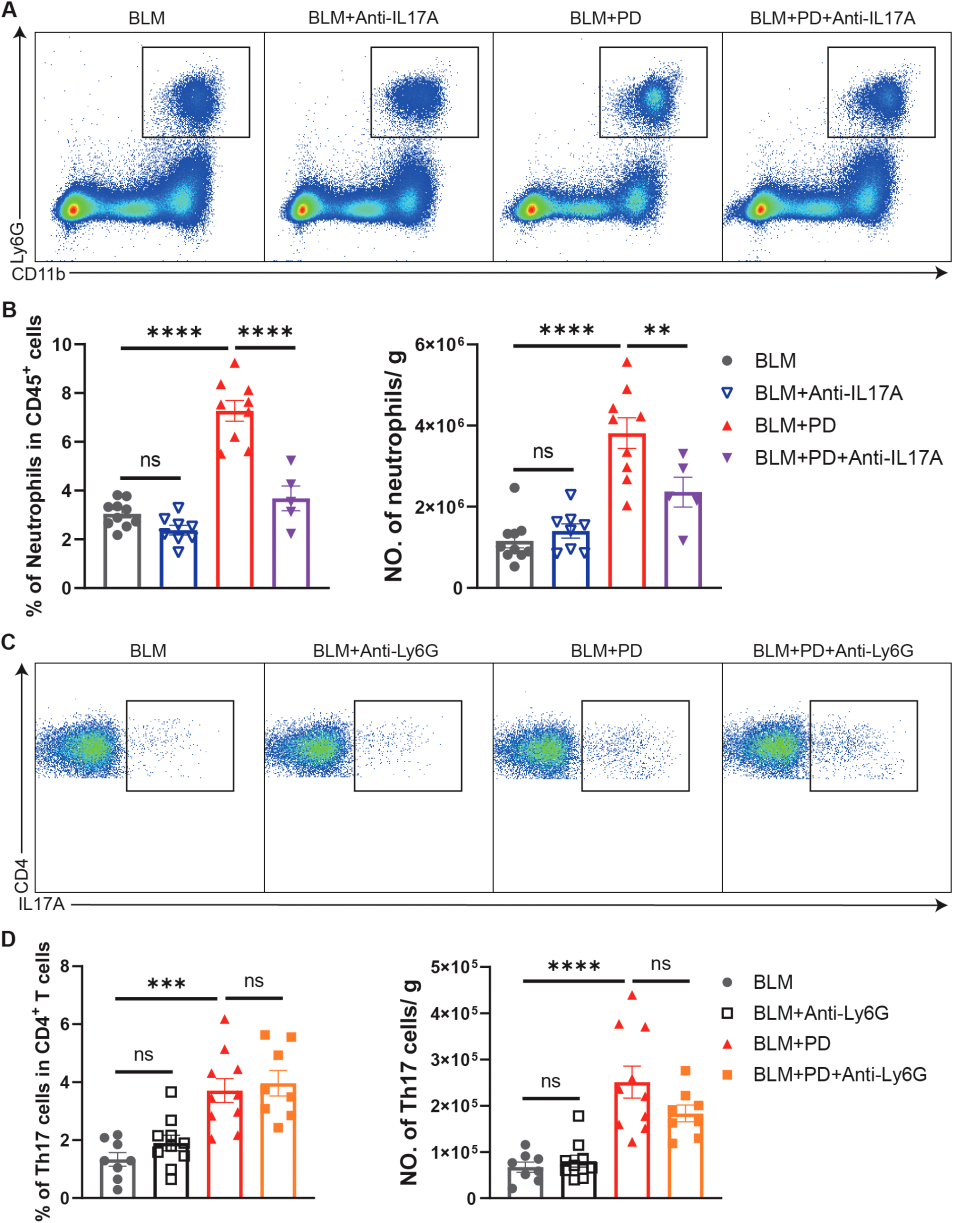
Th17 cells regulate neutrophils via IL-17A in PD-induced aggravation of pulmonary fibrosis. **(A)** Representative flow cytometry plots of neutrophils in mouse lungs at 7 days after BLM administration. **(B)** Quantifications of neutrophils in mouse lungs as percentages and numbers. NO., number. **(C)** Representative flow cytometry plots of Th17 cells in mouse lungs at 7 days after BLM administration. **(D)** Quantifications of Th17 cells in mouse lungs as percentages and numbers. NO., number. n=5-10. Data are presented as mean ± SEM. One-way ANOVA was used for statistical analysis. ns, not significant; *P < 0.05, **P < 0.01, ***P < 0.001, and ****P < 0.0001.

We next evaluated the relationship between neutrophils and Th17 cells in PD-induced aggravation of pulmonary fibrosis. Neutrophils in the lungs were comparable between BLM-administrated mice and BLM+Anti-IL17A treated mice, while the percentage and number of neutrophils were significantly lower in BLM+PD+anti-IL17A mice than in BLM+PD mice (**Figures 4A, B**). These results indicated that IL-17A was the upstream signal triggering neutrophils to mediate PD-induced aggravation of pulmonary fibrosis. However, Th17 cells in lungs were comparable between the BLM+PD group and BLM+PD+Anti-Ly6G group (**Figures 4C, D**). This result indicated that neutrophils did not influence the number of Th17 cells in mice with pulmonary fibrosis. Hence, these results revealed that neutrophils and Th17 cells are critical for the PD-induced aggravation of pulmonary fibrosis and that Th17 cells regulate neutrophils via IL-17A.

### PD enriches *Pg* in the lung and *Pg* aggravates BLM-induced pulmonary fibrosis in mice

**Figure 5:**
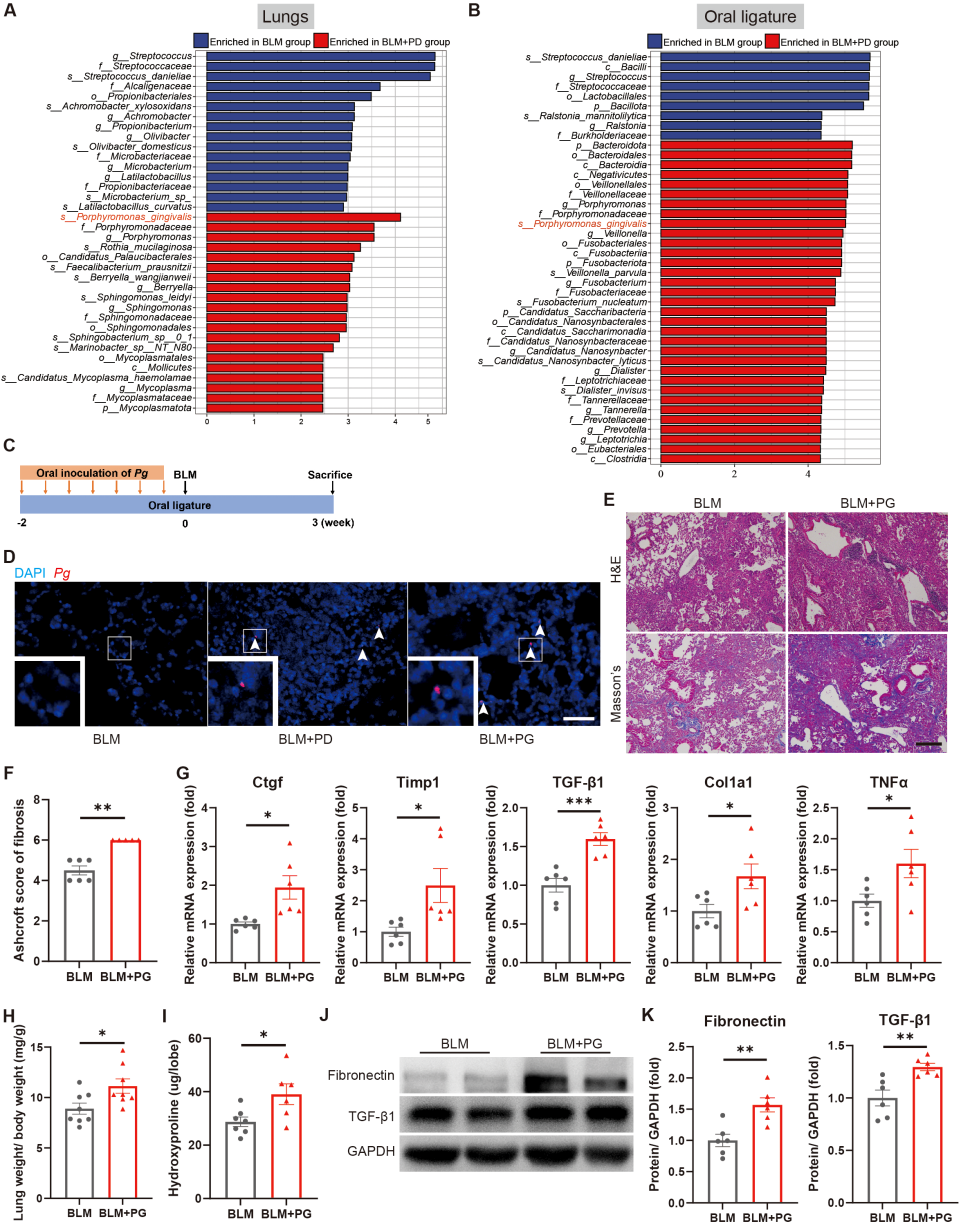
*Porphyromonas gingivalis* (*Pg*) aggravates BLM-induced pulmonary fibrosis in mice. **(A, B)** Linear discriminant analysis effect size (LEfSe) of enriched bacterial species in lungs **(A)** and oral ligatures **(B)** of mice with pulmonary fibrosis and with or without PD by 16S rRNA gene sequencing. n=4:5. **(C)** Schematic illustration of the experimental design. Periodontitis was induced in mice of the PG group by ligation of molars and oral inoculation of *Pg*. **(D)** Representative fluorescence *in situ* hybridization (FISH) for *Pg* in mouse lungs using Alexa Fluor 594-conjugated *Pg* probe. Boxed areas are shown in higher magnification by inset images. Scale bar: 100 µm. **(E)** Representative images of H&E and Masson’s trichrome staining of lung sections of mice with pulmonary fibrosis and with or without *Pg* inoculation. Scale bar: 200 µm. **(F)** Quantitative analysis of pulmonary fibrosis based on Masson’s trichrome staining. **(G)** qRT–PCR analysis of fibrotic genes in mouse lungs. **(H)** Lung weight to body weight ratio of mice. **(I)** Quantification of hydroxyproline content in mouse lungs. **(J–K)** Representative Western blotting analysis **(J)** and quantifications **(K)** of Fibronectin and TGF-β1 in mouse lungs. n=5-8. Data are presented as mean ± SEM. Student’s t-test was used for statistical analysis. *P < 0.05, **P < 0.01, and ***P < 0.001.

Oral microbes were detected in the BALF microbiome of IPF patients, which possibly promoted the occurrence or progression of IPF (Tong et al., 2019). Therefore, we next assessed whether PD-related pathogenic bacteria transferred from the oral cavity to the lung and consequently impacted pulmonary fibrosis. We analysed both the lung microbiome and oral microbiome in the BLM group and BLM+PD group by 16S rRNA gene sequencing. For the lung microbiome, the alpha diversity and beta diversity were not notably different between the BLM+PD group and BLM group (**Supplementary Figure S3A, B**). In contrast, the alpha diversity and beta diversity of the oral microbiome were significantly different between the two groups (**Supplementary Figure S3C, D**). The LEfSe results of the enriched bacterial species in lungs and oral ligatures demonstrated that *Porphyromonas gingivalis* was among the top-ranked PD-related species in the BLM+PD group (**Figures 5A, B**).

To determinate the effect of *Pg* on pulmonary fibrosis in mice, we established a *Pg*-induced PD model by ligation of bilateral maxillary second molars and oral inoculation of *Pg* in the PG group and subsequently administrated BLM (**Figure 5C**). *Pg* was detected in lung sections of mice from both the BLM+PD group and BLM+PG group, which confirmed *Pg* translocating from the oral to the lungs (**Figure 5D**). According to the results of H&E and Masson’s trichrome staining, *Pg* caused more severe injury and significantly increased collagen accumulation in lungs of the mice (**Figures 5E, F**). *Pg* also notably promoted the expression of fibrotic genes (**Figure 5G**) and fibrotic proteins (**Figures 5J, K**). Compared with the BLM group, mice in the BLM+PG group presented markedly heavier lungs and higher levels of hydroxyproline in the lungs (**Figures 5H, I**). Overall, *Pg* translocated from the oral to the lung and aggravated BLM-induced pulmonary fibrosis in mice.

### *Pg* increases the infiltration of neutrophils and Th17 cells in mice with pulmonary fibrosis

**Figure 6:**
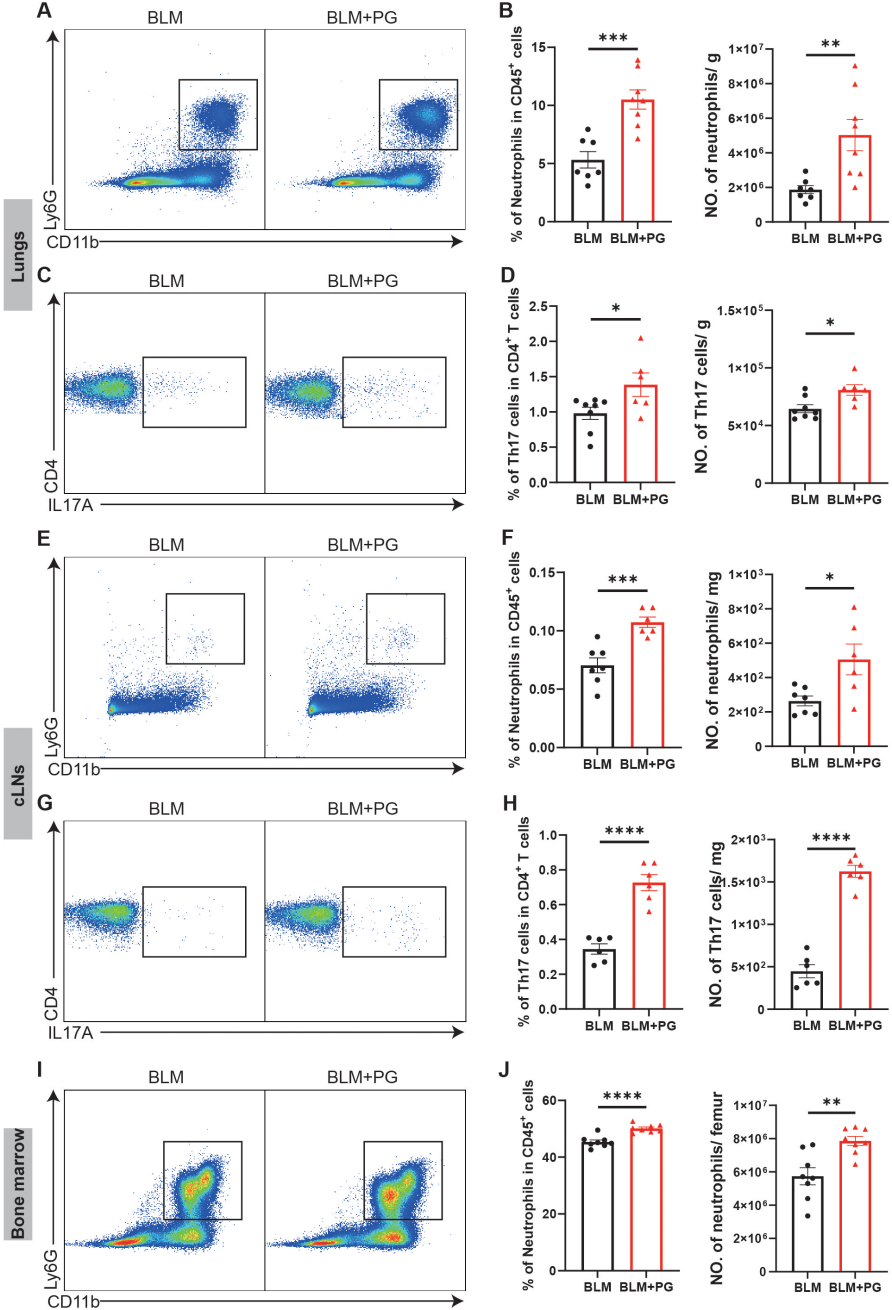
*Pg* increases the infiltration of neutrophils and Th17 cells in mice with pulmonary fibrosis. **(A)** Representative flow cytometry plots of neutrophils in mouse lungs at 7 days after BLM administration. Periodontitis was induced in mice of the PG group by ligation of molars and oral inoculation of *Pg*. **(B)** Quantifications of neutrophils in mouse lungs as percentages and numbers. **(C)** Representative flow cytometry plots of Th17 cells in mouse lungs at 7 days after BLM administration. **(D)** Quantifications of Th17 cells in mouse lungs as percentages and numbers. **(E)** Representative flow cytometry plots of neutrophils in mouse cLNs at 7 days after BLM administration. **(F)** Quantifications of neutrophils in mouse cLNs as percentages and numbers. **(G)** Representative flow cytometry plots of Th17 cells in mouse cLNs at 7 days after BLM administration. **(H)** Quantifications of Th17 cells in mouse cLNs as percentages and numbers. **(I)** Representative flow cytometry plots of neutrophils in mouse bone marrow at 7 days after BLM administration. **(J)** Quantifications of neutrophils in mouse bone marrow as percentages and numbers. n=6-8. Data are presented as mean ± SEM. Student’s t-test was used for statistical analysis. *P < 0.05, **P < 0.01, ***P < 0.001, and ****P < 0.0001.

To explore immune mechanisms underlying *Pg*-driven exacerbation of pulmonary fibrosis, we performed flow cytometry analysis of the lungs, cLNs and bone marrow at 7 days after BLM administration, which was an early inflammation stage that determines the following development of pulmonary fibrosis. Consistent with the PD-induced infiltration of neutrophils and Th17 cells (**Figure 2**), *Pg* also significantly increased neutrophils and Th17 cells in lungs of mice (**Figures 6A–D**). The percentages and numbers of other myeloid cells and lymphoid cells did not exhibit the same significant changes between the two groups (**Supplementary Figure S4A–C**). We further analysed immune cells in cLNs and the bone marrow in an unbiased manner. In line with subgingival plaque-induced PD, *Pg* also caused notable increases of neutrophils and Th17 cells in cLNs (**Figures 6E–H**). Additionally, *Pg* notably increased the percentage and number of B cells in cLNs (**Supplementary Figures S4D–F**). Compared with the BLM group, the percentage and number of neutrophils in the bone marrow of the BLM+PG group were greater (**Figures 6I, J**). To some extent, *Pg* also affected other myeloid cells and lymphoid cells in the bone marrow (**Supplementary Figures S4G, H**). Taken together, these results illustrated that *Pg* promoted the expansion of neutrophils and Th17 cells in lungs, cLNs and bone marrow of mice with BLM-induced pulmonary fibrosis.

## Discussion

**Figure 7:**
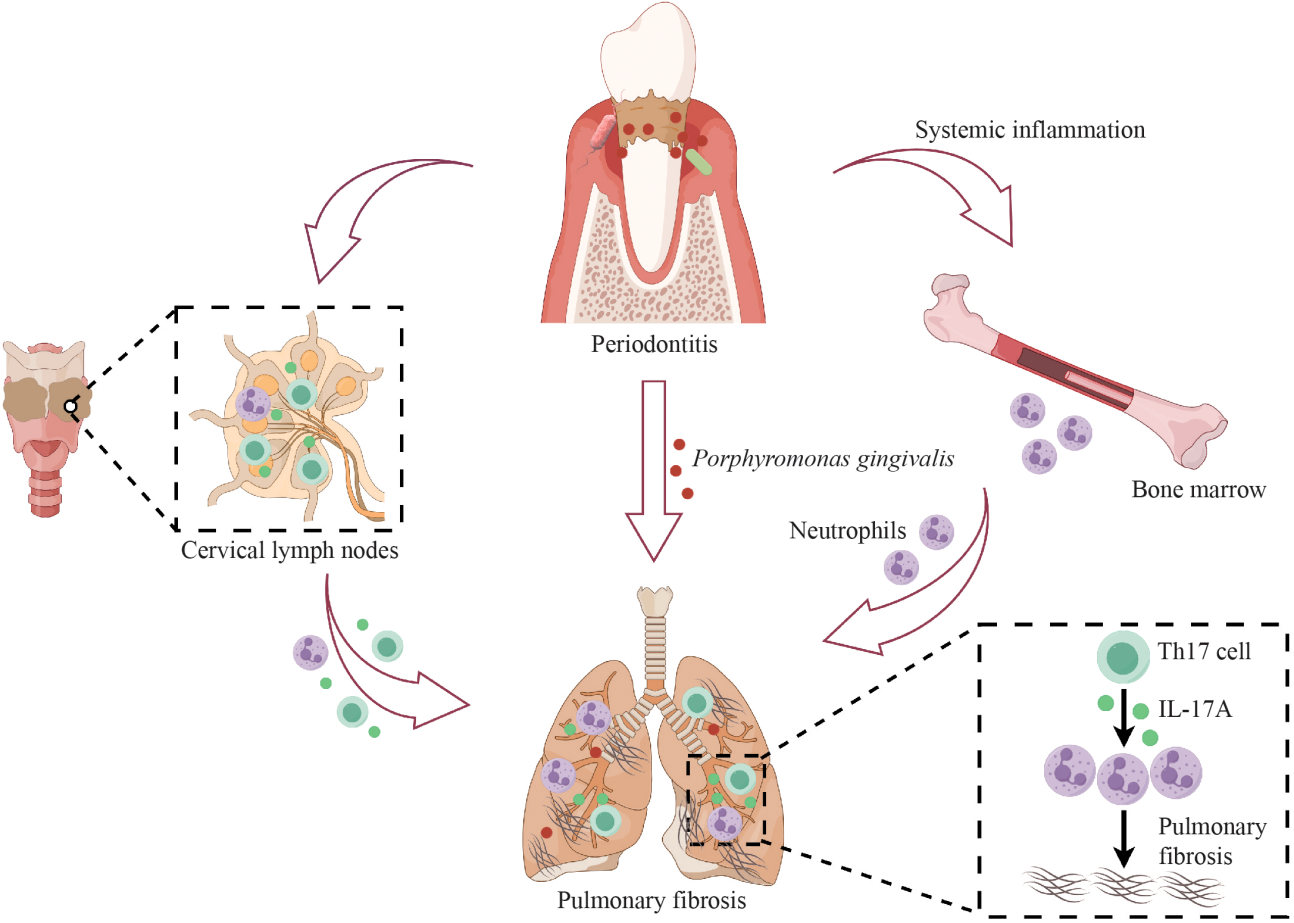
Schematic illustration of the mechanisms by which periodontitis (PD) aggravates pulmonary fibrosis. PD aggravates BLM-induced pulmonary fibrosis by promoting the infiltration of neutrophils and Th17 cells in the lungs, and Th17 cells regulate neutrophils via IL-17A in PD-induced aggravation of pulmonary fibrosis. PD facilitates the translocation of the oral pathogen, *Porphyromonas gingivalis*, to the lung and *Porphyromonas gingivalis* also induces the expansion of Th17 cells and neutrophils and aggravates pulmonary fibrosis. This figure was created with the assistance of Figdraw.

Several clinical studies have reported that oral microbiota was possibly a risk factor for pulmonary fibrosis (Molyneaux et al., 2014; Tong et al., 2019; Invernizzi et al., 2021). As a very prevalent oral infectious disease, PD causes oral microbiota dysbiosis and notably increases oral pathogens (Kinane et al., 2017). However, there is a lack of identification and mechanistic exploration of the relationships among PD, oral microbiota and pulmonary fibrosis. Here, our results demonstrated that PD aggravated BLM-induced pulmonary fibrosis in mice and that the ectopically colonized PD pathogen *Pg* promoted the infiltration of neutrophils and Th17 cells, and Th17 cells regulated neutrophils via IL-17A to exacerbate pulmonary fibrosis (**Figure 7**).

Our results revealed an association between PD and IPF, and verified that PD exacerbates BLM-induced pulmonary fibrosis in mice. PD caused more severe lung damage and obviously increased collagen accumulation in lungs, heavier lungs as well as higher levels of hydroxyproline. PD also promoted the mRNA and protein levels of fibrotic markers. Our models used in this study mimicked those in clinical practice to some extent and provided more insights into the causal relationship between PD and the progression of pulmonary fibrosis. Subgingival plaques from PD patients were orally inoculated into mice to simulate the oral microbiome of human patients (Zhou et al., 2023b). The BLM model is acknowledged as the most extensively applied experimental model in IPF research (Tashiro et al., 2017). The administration of BLM first led to excessive inflammatory infiltrates and subsequent extracellular matrix accumulation, ultimately resulting in pulmonary fibrosis (Della Latta et al., 2015). PD leads to systemic chronic inflammation, which may partially explain why PD exacerbated pulmonary fibrosis in our study.

To reveal the mechanisms by which PD aggravated pulmonary fibrosis, we next performed flow cytometry analysis and found that PD increased the infiltration of neutrophils and Th17 cells. PD expanded neutrophils and Th17 cells before BLM administration (0 day), during the inflammation period (7 days) and fibrosis period (21 days). These results were same as previous studies revealing that PD leads to chronically deregulated inflammatory conditions and impacts the systemic immune response (Albuquerque-Souza and Sahingur, 2022). In our study, PD promoted the expansion of neutrophils and Th17 cells in lungs even before BLM administration. Both neutrophils and Th17 cells were determined vital for exacerbating pulmonary fibrosis (Aoki et al., 2005; Yang et al., 2019). Our results showed that PD markedly increased neutrophils and Th17 cells in the cLNs and neutrophils in the bone marrow. Consistent with previous studies, PD significantly enriched neutrophils and Th17 cells in draining cLNs (Papadakou et al., 2017; Zhou et al., 2023a). PD induces a sustained increase in myelopoiesis and the production of neutrophils in the bone marrow (Li et al., 2022). The bone marrow generates neutrophils and then releases to peripheral organs, including lungs (Summers et al., 2010). The lymphatic circulatory system enables leukocyte trafficking among draining lymph nodes in response to inflammation (Au et al., 2002). Hence, these local and systemic immune disorders caused by PD further promote the progression of pulmonary fibrosis.

In our study, neutrophils and Th17 cells were demonstrated indispensable for PD-induced exacerbation of pulmonary fibrosis, and Th17 cells regulated neutrophils via IL-17A to aggravate pulmonary fibrosis. Various studies have confirmed the importance of neutrophils, Th17 cells and IL-17A in pulmonary fibrosis. These studies verified that neutrophil or IL-17A depletion attenuated BLM-induced pulmonary inflammation and pulmonary fibrosis (Lei et al., 2016; Leslie et al., 2020; Chen et al., 2022). Reasonably, our study showed that PD did not aggravate pulmonary fibrosis after neutrophil depletion or IL-17A depletion. IL-17A is generated mainly by Th17 cells (Mills, 2023) and has been demonstrated to promote immune cells like macrophages and neutrophils to aggravate airway inflammation (Sergejeva et al., 2005; Cheung et al., 2008; Vanaudenaerde et al., 2011). Th17 cells reportedly play important roles in neutrophil recruitment and aggregation in the lungs by secreting IL-17A and increasing the expression of chemokines (Liu et al., 2015; Yang et al., 2022). IL-17A acted on many nonimmune cells and prompted them to release chemokines, which were sensed by neutrophils in blood vessels and neutrophils then migrated to the focus (Knochelmann et al., 2018). Additionally, by upregulating G-CSF expression, IL-17A accelerated proliferation and differentiation of neutrophil precursors, and the release of mature neutrophils from bone marrow (Fan et al., 2023). Consistent with these studies, our results also verified that Th17 cells assembled neutrophils via IL-17A in the lung to promote PD-induced aggravation of pulmonary fibrosis.

Healthy lungs are traditionally thought to be sterile, but more and more studies have detected the microbiota in the lungs via advanced sequencing methods in recent years (Whiteside et al., 2021). Many pulmonary diseases are strongly associated with lung dysbiosis or specific microbiota (Durack et al., 2017; Wang et al., 2021). Pathological changes in the lung structure and damaged mucus clearance because of lung diseases probably contribute to microbial dysbiosis and microbial colonization, which in turn might promote the development of lung diseases by upregulating or downregulating inflammatory signals (Natalini et al., 2023). The oral microbiota is acknowledged as the primary source of the lung microbiota, and the lung microbiota resembles the oral microbiota more than microbiota of other body sites (Venkataraman et al., 2015; Dickson et al., 2016). Greater oral microbial diversity was reported to be related to more severe conditions and a greater risk of death in IPF patients (O’Dwyer et al., 2024). A previous study showed that most translocations (32.84%) of the lung microbiome in IPF patients were from the oral microbiome (Tong et al., 2019). As an entrance of the respiratory tract, oral cavity is anatomically connected with the respiratory system. Provided with the proximity, oral microbiota including periodontal pathogens have more chance to enter the trachea and lung. Oral microbiota is easily translocated to the lung by daily inhalation, coughing and swallowing (Soussan et al., 2019). For those patients who need mechanical ventilation, oral microbiota migrates rapidly from the oral cavity and upper respiratory tract into the lung when performing orotracheal intubation (de Carvalho Baptista et al., 2018). Moreover, the oral cavity turns dry when the patient is bedridden for extended periods. This dry oral environment promotes oropharyngeal bacterial colonization and impaired swallowing function of patients further leads to bacterial stagnation in the pharyngeal cavity, which contributes to the aspiration of oral microbiota to the lung (Rehm et al., 2010). Periodontal pockets are prone to ulceration and bleeding, and therefore periodontal pathogens can also disseminate to the lung by blood (Forner et al., 2006). Besides, conditions like long-term smoking, diabetes and nasal intubation impairs the body immune system, which facilitate the translocation of oral microbiota to the lung (Pathak et al., 2021).

Dysregulated lung bacteria are reported to motivate the expression of neutrophil-recruiting genes and Th17 cell-promoting genes via their outer membrane vesicles to aggravate lung fibrosis in BLM-administrated mice (Yang et al., 2019). Furthermore, it has been reported that subjects with the four periodontal pathogens, *Tannerella forsythia*, *Pg*, *Aggregatibacter actinomycetemcomitans* and *Treponema denticola* in the lung suffered from PD more frequently than those subjects without these periodontal pathogens in the lung. The level of inflammatory aMMP8 tended to be higher in the bronchial fluid in subjects with the four periodontal pathogens. This indicates that PD possibly increases the risk of pulmonary colonization of periodontal pathogens, and such colonization seems to be related with increased lung inflammation (Schmidlin et al., 2015). As a classic pathogen of PD, *Pg* is broadly acknowledged to be related to pneumonia, asthma, chronic obstructive pulmonary disease and other respiratory diseases and is found in the lungs or BALF from patients (Shi et al., 2023). *Pg*-induced PD resulted in more obvious pulmonary inflammation and increased neutrophils in the peripheral blood and lungs, as well as the presence of *Pg* gingipains in lung tissues (Tian et al., 2022). Outer membrane vesicles of *Pg* were reported to induce cell death by disrupting the barrier system in lung epithelial cells (He et al., 2020). Intratracheal injection of *Pg* culture supernatant significantly exacerbated pneumonia by increasing the production of TNF-α and IL-17, which indicates the importance of virulence factors produced by *Pg* (Okabe et al., 2021). Additionally, *Pg* also led to a significant increase of Th17 cells in cLNs (Sandal et al., 2016). Consistent with these previous results, our study verified that PD contributed to ectopic colonization of *Pg* in the lung, and *Pg* increased the infiltration of neutrophils and Th17 cells in mice.

We acknowledge several limitations in this study. Although we verified that PD aggravated BLM-induced pulmonary fibrosis in mice, we do not know the causal relationship between PD and IPF in humans. In the future, researchers can investigate the effect of PD on IPF and collect clinical samples to explore changes of lung microbiota by clinical studies. Besides, we revealed that *Pg*-induced PD exacerbated pulmonary fibrosis by promoting the infiltration of neutrophils and Th17 cells in mice, but the direct effects of *Pg* and *Pg* related virulence factors on the lung remained unknown. These underlying mechanisms can be explored in future experiments.

## Conclusion

The present study demonstrated that PD aggravated BLM-induced pulmonary fibrosis in mice. Mechanistically, PD promoted ectopic colonization of *Pg* in lungs and markedly increased the infiltration of neutrophils and Th17 cells in mice, thereby exacerbating inflammation and subsequent pulmonary fibrosis. Furthermore, our data verified that Th17 cells recruited neutrophils via IL-17A to aggravate pulmonary fibrosis. Together, these data provide insights into the correlation between PD and IPF, reveal novel mechanisms underlying this correlation, and suggest treatment targeting PD or *Pg* as a promising strategy for clinically ameliorating IPF.

## Data Availability

The data that support the findings of this study are available in the NCBI Sequence Read Archive database with the accession number PRJNA1138471.

## References

[B1] Abe T. Hajishengallis G. (2013). Optimization of the ligature-induced periodontitis model in mice. J. Immunol. Methods 394, 49–54. doi: 10.1016/j.jim.2013.05.002 23672778 PMC3707981

[B2] Albuquerque-Souza E. Sahingur S. E. (2022). Periodontitis, chronic liver diseases, and the emerging oral-gut-liver axis. Periodontol 2000 89, 125–141. doi: 10.1111/prd.12427 35244954 PMC9314012

[B3] Amati F. Stainer A. Mantero M. Gramegna A. Simonetta E. Suigo G.. (2022). Lung microbiome in idiopathic pulmonary fibrosis and other interstitial lung diseases. Int. J. Mol. Sci. 23. doi: 10.3390/ijms23020977 PMC8779068 35055163

[B4] Aoki F. Kurabayashi M. Hasegawa Y. Kojima I. (2005). Attenuation of bleomycin-induced pulmonary fibrosis by follistatin. Am. J. Respir. Crit. Care Med. 172, 713–720. doi: 10.1164/rccm.200412-1620OC 15976370

[B5] Au B. McCulloch C. A. Hay J. B. (2002). Quantitative studies on the movement of fluid and lymphocytes through periodontal tissue and into the draining lymph. Microsc Res. Tech 56, 66–71. doi: 10.1002/jemt.10004 11810708

[B6] Bai L. Chen B. Y. Liu Y. Zhang W. C. Duan S. Z. (2022). A mouse periodontitis model with humanized oral bacterial community. Front. Cell Infect. Microbiol 12. doi: 10.3389/fcimb.2022.842845 PMC8902145 35273925

[B7] Bassis C. M. Erb-Downward J. R. Dickson R. P. Freeman C. M. Schmidt T. M. Young V. B.. (2015). Analysis of the upper respiratory tract microbiotas as the source of the lung and gastric microbiotas in healthy individuals. mBio 6, e00037. doi: 10.1128/mBio.00037-15 25736890 PMC4358017

[B8] Bonella F. Spagnolo P. Ryerson C. (2023). Current and future treatment landscape for idiopathic pulmonary fibrosis. Drugs 83, 1581–1593. doi: 10.1007/s40265-023-01950-0 37882943 PMC10693523

[B9] Chen S. Zhang X. Yang C. Wang S. Shen H. (2022). Essential role of IL-17 in acute exacerbation of pulmonary fibrosis induced by non-typeable Haemophilus influenzae. Theranostics 12, 5125–5137. doi: 10.7150/thno.74809 35836804 PMC9274745

[B10] Cheung P. F. Wong C. K. Lam C. W. (2008). Molecular mechanisms of cytokine and chemokine release from eosinophils activated by IL-17A, IL-17F, and IL-23: implication for Th17 lymphocytes-mediated allergic inflammation. J. Immunol. 180, 5625–5635. doi: 10.4049/jimmunol.180.8.5625 18390747

[B11] Coffey N. O’Leary F. Burke F. Kirwan L. O’Regan P. Plant B.. (2024). Periodontal disease prevalence and oral hygiene status of adults with cystic fibrosis: A case-control study. J. Clin. Periodontol 51, 571–582. doi: 10.1111/jcpe.13944 38233039

[B12] de Carvalho Baptista I. M. Martinho F. C. Nascimento G. G. da Rocha Santos C. E. Prado R. F. D. Valera M. C. (2018). Colonization of oropharynx and lower respiratory tract in critical patients: Risk of ventilator-associated pneumonia. Arch. Biol. 85, 64–69. doi: 10.1016/j.archoralbio.2017.09.029 29031240

[B13] Della Latta V. Cecchettini A. Del Ry S. Morales M. A. (2015). Bleomycin in the setting of lung fibrosis induction: From biological mechanisms to counteractions. Pharmacol. Res. 97, 122–130. doi: 10.1016/j.phrs.2015.04.012 25959210

[B14] Dickson R. P. Erb-Downward J. R. Martinez F. J. Huffnagle G. B. (2016). The microbiome and the respiratory tract. Annu. Rev. Physiol. 78, 481–504. doi: 10.1146/annurev-physiol-021115-105238 26527186 PMC4751994

[B15] Durack J. Lynch S. V. Nariya S. Bhakta N. R. Beigelman A. Castro M.. (2017). Features of the bronchial bacterial microbiome associated with atopy, asthma, and responsiveness to inhaled corticosteroid treatment. J. Allergy Clin. Immunol. 140, 63–75. doi: 10.1016/j.jaci.2016.08.055 27838347 PMC5502827

[B16] Fan X. Shu P. Wang Y. Ji N. Zhang D. (2023). Interactions between neutrophils and T-helper 17 cells. Front. Immunol. 14. doi: 10.3389/fimmu.2023.1279837 PMC10619153 37920459

[B17] Feng N. Han X. Peng D. Geng F. Li Q. Pan C.. (2024). P. gingivalis alters lung microbiota and aggravates disease severity of COPD rats by up-regulating Hsp90α/MLKL. J. Microbiol 16, 2334588. doi: 10.1080/20002297.2024.2334588 PMC10977012 38550659

[B18] Forner L. Larsen T. Kilian M. Holmstrup P. (2006). Incidence of bacteremia after chewing, tooth brushing and scaling in individuals with periodontal inflammation. J. Clin. Periodontol 33, 401–407. doi: 10.1111/j.1600-051X.2006.00924.x 16677328

[B19] Gaeckle N. T. Pragman A. A. Pendleton K. M. Baldomero A. K. Criner G. J. (2020). The oral-lung axis: the impact of oral health on lung health. Respir. Care 65, 1211–1220. doi: 10.4187/respcare.07332 32156792

[B20] Hajishengallis G. Chavakis T. (2021). Local and systemic mechanisms linking periodontal disease and inflammatory comorbidities. Nat. Rev. Immunol. 21, 426–440. doi: 10.1038/s41577-020-00488-6 33510490 PMC7841384

[B21] He Y. Shiotsu N. Uchida-Fukuhara Y. Guo J. Weng Y. Ikegame M.. (2020). Outer membrane vesicles derived from Porphyromonas gingivalis induced cell death with disruption of tight junctions in human lung epithelial cells. Arch. Biol. 118, 104841. doi: 10.1016/j.archoralbio.2020.104841 32717445

[B22] Hübner R. H. Gitter W. El Mokhtari N. E. Mathiak M. Both M. Bolte H.. (2008). Standardized quantification of pulmonary fibrosis in histological samples. Biotechniques 44, 514–507. doi: 10.2144/000112729 18476815

[B23] Invernizzi R. Wu B. G. Barnett J. Ghai P. Kingston S. Hewitt R. J.. (2021). The respiratory microbiome in chronic hypersensitivity pneumonitis is distinct from that of idiopathic pulmonary fibrosis. Am. J. Respir. Crit. Care Med. 203, 339–347. doi: 10.1164/rccm.202002-0460OC 32692582 PMC7874329

[B24] Jungblut M. Oeltze K. Zehnter I. Hasselmann D. Bosio A. (2009). Standardized preparation of single-cell suspensions from mouse lung tissue using the gentleMACS Dissociator. J. Vis Exp. 29). doi: 10.3791/1266 PMC2798855 19574953

[B25] Karampitsakos T. Juan-Guardela B. M. Tzouvelekis A. Herazo-Maya J. D. (2023). Precision medicine advances in idiopathic pulmonary fibrosis. EBioMedicine 95, 104766. doi: 10.1016/j.ebiom.2023.104766 37625268 PMC10469771

[B26] Kinane D. F. Stathopoulou P. G. Papapanou P. N. (2017). Periodontal diseases. Nat. Rev. Dis. Primers 3, 17038. doi: 10.1038/nrdp.2017.38 28805207

[B27] Knochelmann H. M. Dwyer C. J. Bailey S. R. Amaya S. M. Elston D. M. Mazza-McCrann J. M.. (2018). When worlds collide: Th17 and Treg cells in cancer and autoimmunity. Cell Mol. Immunol. 15, 458–469. doi: 10.1038/s41423-018-0004-4 29563615 PMC6068176

[B28] Koyanagi T. Sakamoto M. Takeuchi Y. Maruyama N. Ohkuma M. Izumi Y. (2013). Comprehensive microbiological findings in peri-implantitis and periodontitis. J. Clin. Periodontol 40, 218–226. doi: 10.1111/jcpe.12047 23294017

[B29] Kumar P. S. Griffen A. L. Moeschberger M. L. Leys E. J. (2005). Identification of candidate periodontal pathogens and beneficial species by quantitative 16S clonal analysis. J. Clin. Microbiol 43, 3944–3955. doi: 10.1128/jcm.43.8.3944-3955.2005 16081935 PMC1233920

[B30] Lei L. Zhao C. Qin F. He Z. Y. Wang X. Zhong X. N. (2016). Th17 cells and IL-17 promote the skin and lung inflammation and fibrosis process in a bleomycin-induced murine model of systemic sclerosis. Clin. Exp. Rheumatol 34 Suppl 100, 14–22. 26750756

[B31] Leslie J. Millar B. J. Del Carpio Pons A. Burgoyne R. A. Frost J. D. Barksby B. S.. (2020). FPR-1 is an important regulator of neutrophil recruitment and a tissue-specific driver of pulmonary fibrosis. JCI Insight 5. doi: 10.1172/jci.insight.125937 PMC7101152 32102985

[B32] Li Q. Wang H. Tan L. Zhang S. Lin L. Tang X.. (2021). Oral Pathogen Fusobacterium nucleatum Coaggregates With Pseudomonas aeruginosa to Modulate the Inflammatory Cytotoxicity of Pulmonary Epithelial Cells. Front. Cell Infect. Microbiol 11. doi: 10.3389/fcimb.2021.643913 PMC8017200 33816348

[B33] Li X. Wang H. Yu X. Saha G. Kalafati L. Ioannidis C.. (2022). Maladaptive innate immune training of myelopoiesis links inflammatory comorbidities. Cell 185, 1709–1727.e1718. doi: 10.1016/j.cell.2022.03.043 35483374 PMC9106933

[B34] Liu Y. Zeng M. Liu Z. (2015). Th17 response and its regulation in inflammatory upper airway diseases. Clin. Exp. Allergy 45, 602–612. doi: 10.1111/cea.12378 25048954

[B35] Meyle J. Chapple I. (2015). Molecular aspects of the pathogenesis of periodontitis. Periodontol 2000 69, 7–17. doi: 10.1111/prd.12104 26252398

[B36] Mills K. H. G. (2023). IL-17 and IL-17-producing cells in protection versus pathology. Nat. Rev. Immunol. 23, 38–54. doi: 10.1038/s41577-022-00746-9 35790881 PMC9255545

[B37] Molyneaux P. L. Cox M. J. Willis-Owen S. A. Mallia P. Russell K. E. Russell A. M.. (2014). The role of bacteria in the pathogenesis and progression of idiopathic pulmonary fibrosis. Am. J. Respir. Crit. Care Med. 190, 906–913. doi: 10.1164/rccm.201403-0541OC 25184687 PMC4299577

[B38] Moss B. J. Ryter S. W. Rosas I. O. (2022). Pathogenic mechanisms underlying idiopathic pulmonary fibrosis. Annu. Rev. Pathol. 17, 515–546. doi: 10.1146/annurev-pathol-042320-030240 34813355

[B39] Natalini J. G. Singh S. Segal L. N. (2023). The dynamic lung microbiome in health and disease. Nat. Rev. Microbiol 21, 222–235. doi: 10.1038/s41579-022-00821-x 36385637 PMC9668228

[B40] Niu C. Lv W. Zhu X. Dong Z. Yuan K. Jin Q.. (2024). Intestinal translocation of live porphyromonas gingivalis drives insulin resistance. J. Dent. Res. 103, 197–207. doi: 10.1177/00220345231214195 38185909

[B41] O’Dwyer D. N. Ashley S. L. Gurczynski S. J. Xia M. Wilke C. Falkowski N. R.. (2019). Lung microbiota contribute to pulmonary inflammation and disease progression in pulmonary fibrosis. Am. J. Respir. Crit. Care Med. 199, 1127–1138. doi: 10.1164/rccm.201809-1650OC 30789747 PMC6515865

[B42] O’Dwyer D. N. Kim J. S. Ma S. F. Ranjan P. Das P. Lipinski J. H.. (2024). Commensal oral microbiota, disease severity, and mortality in fibrotic lung disease. Am. J. Respir. Crit. Care Med. 209, 1101–1110. doi: 10.1164/rccm.202308-1357OC 38051927 PMC11092942

[B43] Okabe T. Kamiya Y. Kikuchi T. Goto H. Umemura M. Suzuki Y.. (2021). Porphyromonas gingivalis Components/Secretions Synergistically Enhance Pneumonia Caused by Streptococcus pneumoniae in Mice. Int. J. Mol. Sci. 22. doi: 10.3390/ijms222312704 PMC8657795 34884507

[B44] Papadakou P. Bletsa A. Yassin M. A. Karlsen T. V. Wiig H. Berggreen E. (2017). Role of hyperplasia of gingival lymphatics in periodontal inflammation. J. Dent. Res. 96, 467–476. doi: 10.1177/0022034516681762 28081372

[B45] Pathak J. L. Yan Y. Zhang Q. Wang L. Ge L. (2021). The role of oral microbiome in respiratory health and diseases. Respir. Med. 185, 106475. doi: 10.1016/j.rmed.2021.106475 34049183

[B46] Pawlaczyk-Kamieńska T. Śniatała R. Batura-Gabryel H. Borysewicz-Lewicka M. Cofta S. (2019). Periodontal status and subgingival biofilms in cystic fibrosis adults. Pol. J. Microbiol 68, 377–382. doi: 10.33073/pjm-2019-040 31880883 PMC7256727

[B47] Podolanczuk A. J. Thomson C. C. Remy-Jardin M. Richeldi L. Martinez F. J. Kolb M.. (2023). Idiopathic pulmonary fibrosis: state of the art for 2023. Eur. Respir. J. 61. doi: 10.1183/13993003.00957-2022 36702498

[B48] Rehm J. Baliunas D. Borges G. L. Graham K. Irving H. Kehoe T.. (2010). The relation between different dimensions of alcohol consumption and burden of disease: an overview. Addiction 105, 817–843. doi: 10.1111/j.1360-0443.2010.02899.x 20331573 PMC3306013

[B49] Rivas Caldas R. Le Gall F. Revert K. Rault G. Virmaux M. Gouriou S.. (2015). Pseudomonas aeruginosa and periodontal pathogens in the oral cavity and lungs of cystic fibrosis patients: a case-control study. J. Clin. Microbiol 53, 1898–1907. doi: 10.1128/jcm.00368-15 25854483 PMC4432057

[B50] Rudney J. D. Chen R. Sedgewick G. J. (2005). Actinobacillus actinomycetemcomitans, Porphyromonas gingivalis, and Tannerella forsythensis are components of a polymicrobial intracellular flora within human buccal cells. J. Dent. Res. 84, 59–63. doi: 10.1177/154405910508400110 15615877

[B51] Sandal I. Karydis A. Luo J. Prislovsky A. Whittington K. B. Rosloniec E. F.. (2016). Bone loss and aggravated autoimmune arthritis in HLA-DRβ1-bearing humanized mice following oral challenge with Porphyromonas gingivalis. Arthritis Res. Ther. 18, 249. doi: 10.1186/s13075-016-1143-6 27784339 PMC5081677

[B52] Schmidlin P. R. Fachinger P. Tini G. Graber S. Seifert B. Dombrowa S.. (2015). Shared microbiome in gums and the lung in an outpatient population. J. Infect. 70, 255–263. doi: 10.1016/j.jinf.2014.10.005 25445885

[B53] Sergejeva S. Ivanov S. Lötvall J. Lindén A. (2005). Interleukin-17 as a recruitment and survival factor for airway macrophages in allergic airway inflammation. Am. J. Respir. Cell Mol. Biol. 33, 248–253. doi: 10.1165/rcmb.2004-0213OC 15901616

[B54] Shi T. Wang J. Dong J. Hu P. Guo Q. (2023). Periodontopathogens Porphyromonas gingivalis and Fusobacterium nucleatum and Their Roles in the Progression of Respiratory Diseases. Pathogens 12. doi: 10.3390/pathogens12091110 PMC10535846 37764918

[B55] Slots J. (2017). Periodontitis: facts, fallacies and the future. Periodontol 2000 75, 7–23. doi: 10.1111/prd.12221 28758294

[B56] Soussan R. Schimpf C. Pilmis B. Degroote T. Tran M. Bruel C.. (2019). Ventilator-associated pneumonia: The central role of transcolonization. J. Crit. Care 50, 155–161. doi: 10.1016/j.jcrc.2018.12.005 30551046

[B57] Spagnolo P. Kropski J. A. Jones M. G. Lee J. S. Rossi G. Karampitsakos T.. (2021). Idiopathic pulmonary fibrosis: Disease mechanisms and drug development. Pharmacol. Ther. 222, 107798. doi: 10.1016/j.pharmthera.2020.107798 33359599 PMC8142468

[B58] Summers C. Rankin S. M. Condliffe A. M. Singh N. Peters A. M. Chilvers E. R. (2010). Neutrophil kinetics in health and disease. Trends Immunol. 31, 318–324. doi: 10.1016/j.it.2010.05.006 20620114 PMC2930213

[B59] Tashiro J. Rubio G. A. Limper A. H. Williams K. Elliot S. J. Ninou I.. (2017). Exploring animal models that resemble idiopathic pulmonary fibrosis. Front. Med. (Lausanne) 4. doi: 10.3389/fmed.2017.00118 PMC5532376 28804709

[B60] Teles F. Collman R. G. Mominkhan D. Wang Y. (2022). Viruses, periodontitis, and comorbidities. Periodontol 2000 89, 190–206. doi: 10.1111/prd.12435 35244970

[B61] Tian H. Zhang Z. Wang X. Liu W. Wang Z. (2022). Role of experimental periodontitis in inducing pulmonary inflammation in mice. Dis. 28, 2294–2303. doi: 10.1111/odi.13949 34174133

[B62] Tong X. Su F. Xu X. Xu H. Yang T. Xu Q.. (2019). Alterations to the lung microbiome in idiopathic pulmonary fibrosis patients. Front. Cell Infect. Microbiol 9. doi: 10.3389/fcimb.2019.00149 PMC6536613 31165050

[B63] Vanaudenaerde B. M. Verleden S. E. Vos R. De Vleeschauwer S. I. Willems-Widyastuti A. Geenens R.. (2011). Innate and adaptive interleukin-17-producing lymphocytes in chronic inflammatory lung disorders. Am. J. Respir. Crit. Care Med. 183, 977–986. doi: 10.1164/rccm.201007-1196PP 21097694

[B64] Venkataraman A. Bassis C. M. Beck J. M. Young V. B. Curtis J. L. Huffnagle G. B.. (2015). Application of a neutral community model to assess structuring of the human lung microbiome. mBio 6. doi: 10.1128/mBio.02284-14 PMC4324308 25604788

[B65] Wang Z. Locantore N. Haldar K. Ramsheh M. Y. Beech A. S. Ma W.. (2021). Inflammatory endotype-associated airway microbiome in chronic obstructive pulmonary disease clinical stability and exacerbations: A multicohort longitudinal analysis. Am. J. Respir. Crit. Care Med. 203, 1488–1502. doi: 10.1164/rccm.202009-3448OC 33332995 PMC8483235

[B66] Whiteside S. A. McGinniss J. E. Collman R. G. (2021). The lung microbiome: progress and promise. J. Clin. Invest. 131. doi: 10.1172/jci150473 PMC8321564 34338230

[B67] Yang D. Chen X. Wang J. Lou Q. Lou Y. Li L.. (2019). Dysregulated lung commensal bacteria drive interleukin-17B production to promote pulmonary fibrosis through their outer membrane vesicles. Immunity 50, 692–706.e697. doi: 10.1016/j.immuni.2019.02.001 30824326

[B68] Yang L. Zheng Y. Miao Y. M. Yan W. X. Geng Y. Z. Dai Y.. (2022). Bergenin, a PPARγ agonist, inhibits Th17 differentiation and subsequent neutrophilic asthma by preventing GLS1-dependent glutaminolysis. Acta Pharmacol. Sin. 43, 963–976. doi: 10.1038/s41401-021-00717-1 34267342 PMC8975945

[B69] Zhou L. J. Lin W. Z. Liu T. Chen B. Y. Meng X. Q. Li Y. L.. (2023a). Oral pathobionts promote MS-like symptoms in mice. J. Dent. Res. 102, 217–226. doi: 10.1177/00220345221128202 36266965

[B70] Zhou L. J. Lin W. Z. Meng X. Q. Zhu H. Liu T. Du L. J.. (2023b). Periodontitis exacerbates atherosclerosis through Fusobacterium nucleatum-promoted hepatic glycolysis and lipogenesis. Cardiovasc. Res. 119, 1706–1717. doi: 10.1093/cvr/cvad045 36943793

