# Supplementary material for: Periodontitis aggravates pulmonary fibrosis by Porphyromonas gingivalis-promoted infiltration of neutrophils and Th17 cells
Source: Front Cell Infect Microbiol. 2025 May 27;15:1595500. doi: 10.3389/fcimb.2025.1595500 (PMC12149129; doi:10.3389/fcimb.2025.1595500)
Supplement: Supplementary file 1 [file DataSheet1.docx]

**Periodontitis aggravates pulmonary fibrosis by *Porphyromonas gingivalis*-promoted infiltration of neutrophils and Th17 cells**

Hui-Lin Ye ^a,b,1^, Xiao-Qian Meng ^a,b,1^, Huxiao Li ^b,c^, Xiaoli Sun^d^, Wen-Zhen Lin ^b^, Lu-Jun Zhou ^b^, Jun Zhang ^b^, Chenchen Hou ^e^, Shuo Xu ^b^, Bo-Yan Chen ^b^, Che Qiu ^b,c^, Yu-Lin Li ^b^, Yong-Li Wang ^b,f^, Li-Feng Yan ^e^, Sheng-Zhong Duan ^a,b,f,*^

^a^Department of Endodontics, Shanghai Ninth People’s Hospital, College of Stomatology, Shanghai Jiao Tong University School of Medicine, Shanghai, China

^b^Laboratory of Oral Microbiota and Systemic Diseases, Shanghai Ninth People’s Hospital, College of Stomatology, Shanghai Jiao Tong University School of Medicine; National Center for Stomatology; National Clinical Research Center for Oral Diseases; Shanghai Key Laboratory of Stomatology, Shanghai, China

^c^Department of Periodontology, Shanghai Ninth People's Hospital, Shanghai Jiao Tong University School of Medicine, Shanghai, China

^d^Department of Respiratory and Critical Care Medicine, Shanghai Pulmonary Hospital, Tongji University School of Medicine, Shanghai, China

^e^Department of Respiratory and Critical Care Medicine, Shanghai Ninth People’s Hospital, Shanghai Jiao Tong University School of Medicine, Shanghai, China

^f^Stomatology Hospital, School of Stomatology, Zhejiang University School of Medicine, Zhejiang Provincial Clinical Research Center for Oral Diseases, Key Laboratory of Oral Biomedical Research of Zhejiang Province, Cancer Center of Zhejiang University, Engineering Research Center of Oral Biomaterials and Devices of Zhejiang Province, Hangzhou, China.

^*^Correspondence: duansz2008@hotmail.com (S-Z.D.)

^1^Hui-Lin Ye and Xiao-Qian Meng contributed equally to this work.


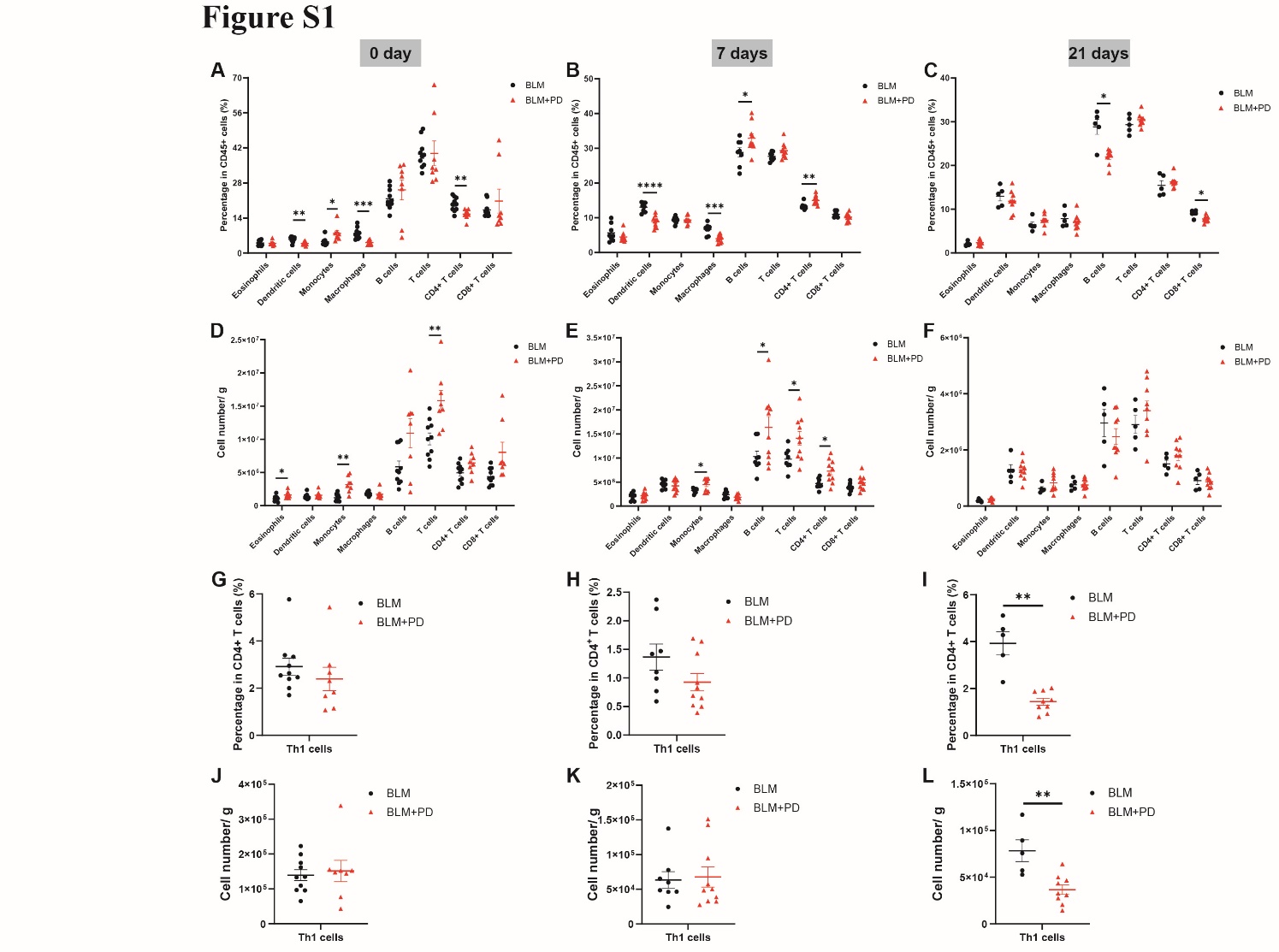


**Fig. S1 Effects of PD on immune cells in lungs of mice with pulmonary fibrosis.** (A-C) Quantifications of immune cells in mouse lungs as percentages of CD45^+^ cells at 0 day (A), 7 days (B) and 21 days (C) post bleomycin challenge. PD was induced in mice by ligation of molars and oral inoculation of subgingival plaques from patients with PD. (D-F) Quantifications of the number of immune cells in mouse lungs at 0 day (D), 7 days (E) and 21 days (F) post bleomycin challenge. (G-I) Quantifications of Th1 cells in mouse lungs as percentages of CD4^+^ T cells at 0 day (G), 7 days (H) and 21 days (I) post bleomycin challenge. (J-L) Quantifications of the number of Th1 cells in mouse lungs at 0 day (J), 7 days (K) and 21 days (L) post bleomycin challenge. n=5-10. Data are presented as mean ± SEM. Student’s t-test was used for statistical analysis. *P < 0.05, **P < 0.01, ***P < 0.001, and ****P < 0.0001.


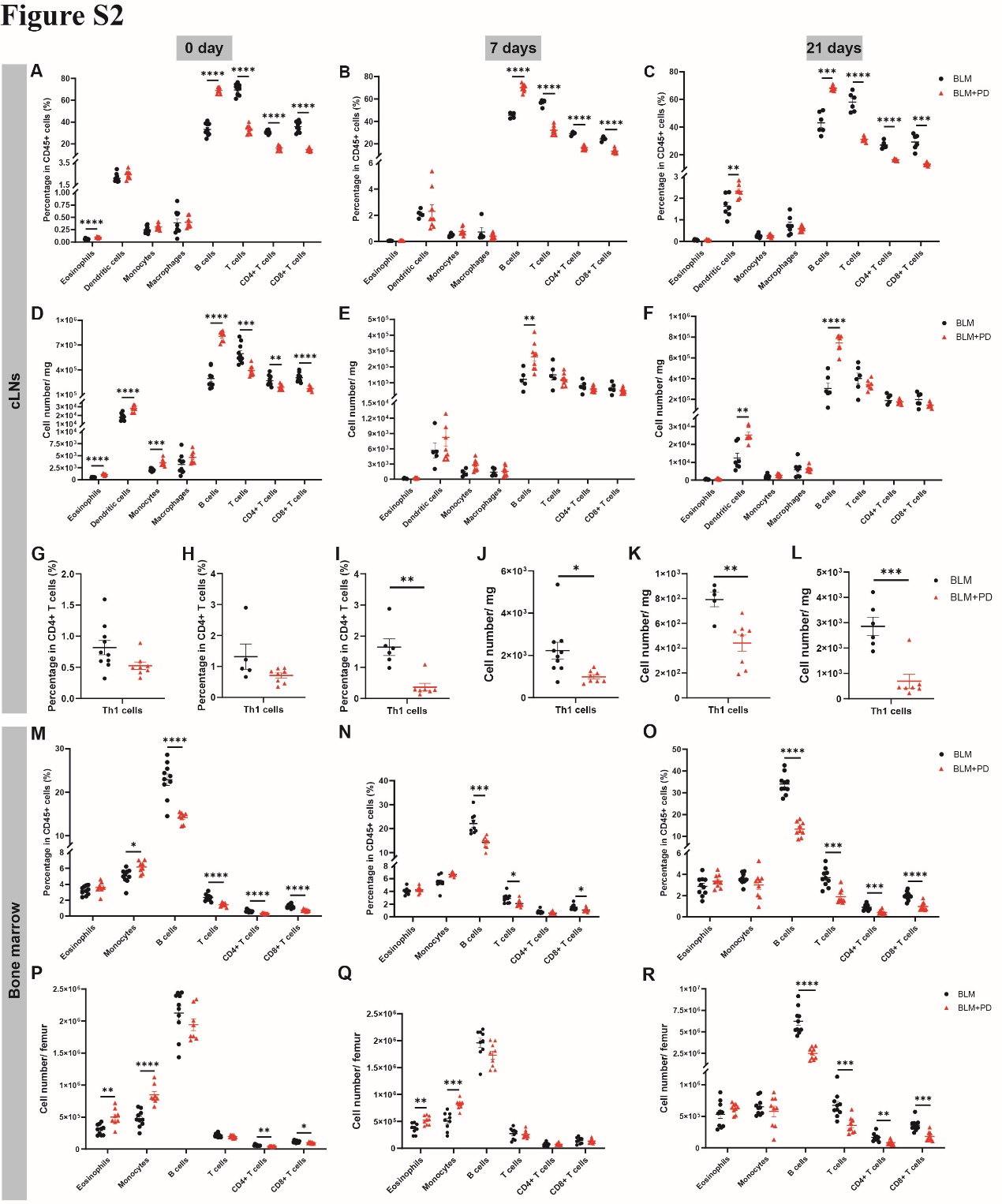


**Fig. S2 Effects of PD on immune cells in cLNs and bone marrow of mice with pulmonary fibrosis.** (A-C) Quantifications of immune cells in mouse cLNs as percentages of CD45^+^ cells at 0 day (A), 7 days (B) and 21 days (C) post bleomycin challenge. (D-F) Quantifications of the number of immune cells in mouse cLNs at 0 day (D), 7 days (E) and 21 days (F) post bleomycin challenge. (G-I) Quantifications of Th1 cells in mouse cLNs as percentages of CD4^+^ T cells at 0 day (G), 7 days (H) and 21 days (I) post bleomycin challenge. (J-L) Quantifications of the number of Th1 cells in mouse cLNs at 0 day (J), 7 days (K) and 21 days (L) post bleomycin challenge. (M-O) Quantifications of immune cells in mouse bone marrow as percentages of CD45^+^ cells at 0 day (M), 7 days (N) and 21 days (O) post bleomycin challenge. (P-R) Quantifications of the number of immune cells in mouse bone marrow at 0 day (P), 7 days (Q) and 21 days (R) post bleomycin challenge. n=5-10. Data are presented as mean ± SEM. Student’s t-test was used for statistical analysis. *P < 0.05, **P < 0.01, ***P < 0.001, and ****P < 0.0001.


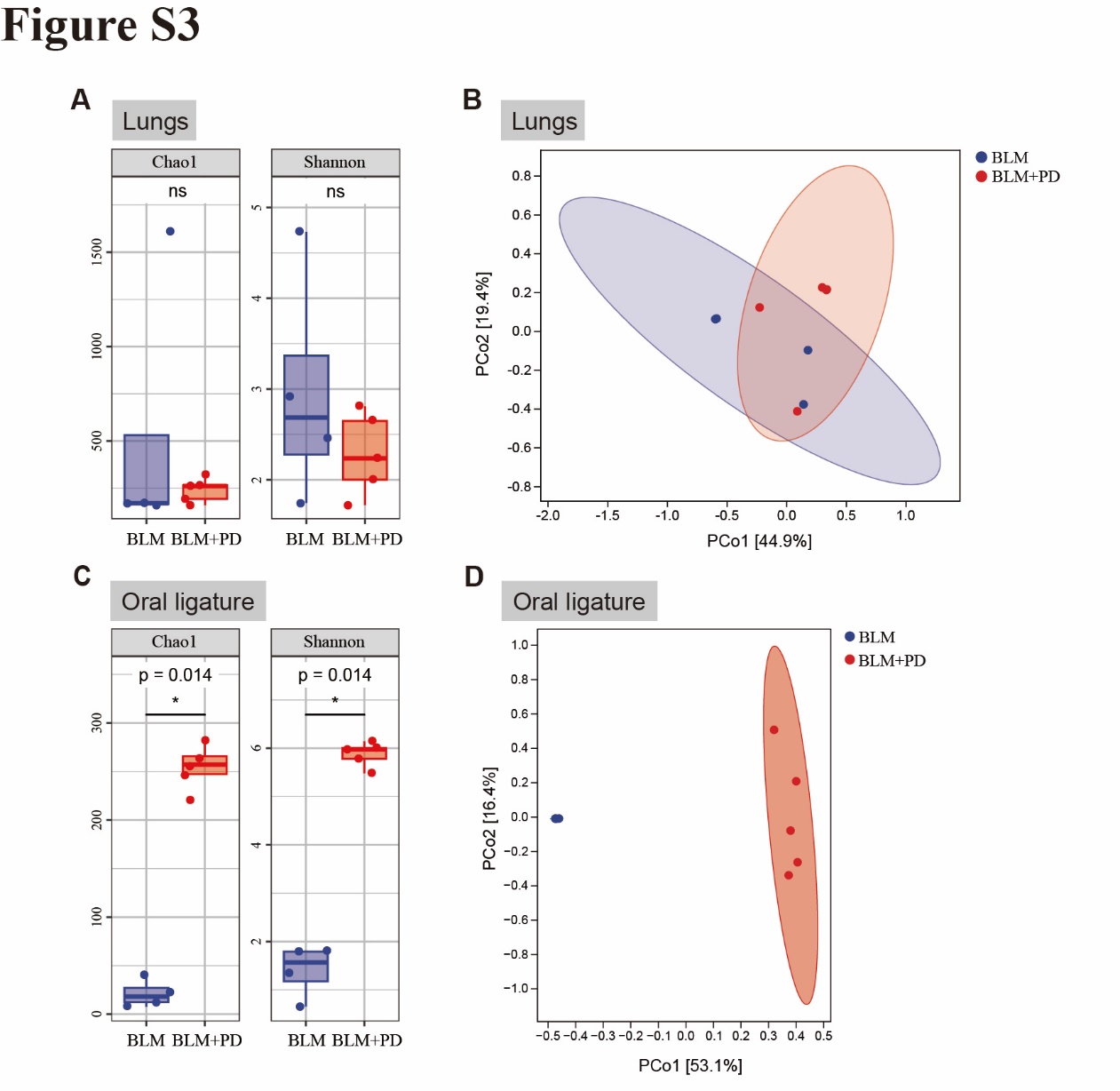


**Fig. S3 Analysis of microbiota in mouse lungs and the oral ligatures.** (A) The α-diversity of microbiota in mouse lungs assessed by Chao1 and Shannon indexes. (B) The β-diversity of microbiota in mouse lungs analyzed by principal coordinate analysis (PCoA). (C) The α-diversity of microbiota in the oral ligatures assessed by Chao1 and Shannon indexes. (D) The β-diversity of microbiota in the oral ligatures analyzed by PCoA. n=4:5. Data are presented as mean ± SEM. Kruskal-Wallis test was used for statistical analysis in A and C. ns, not significant.


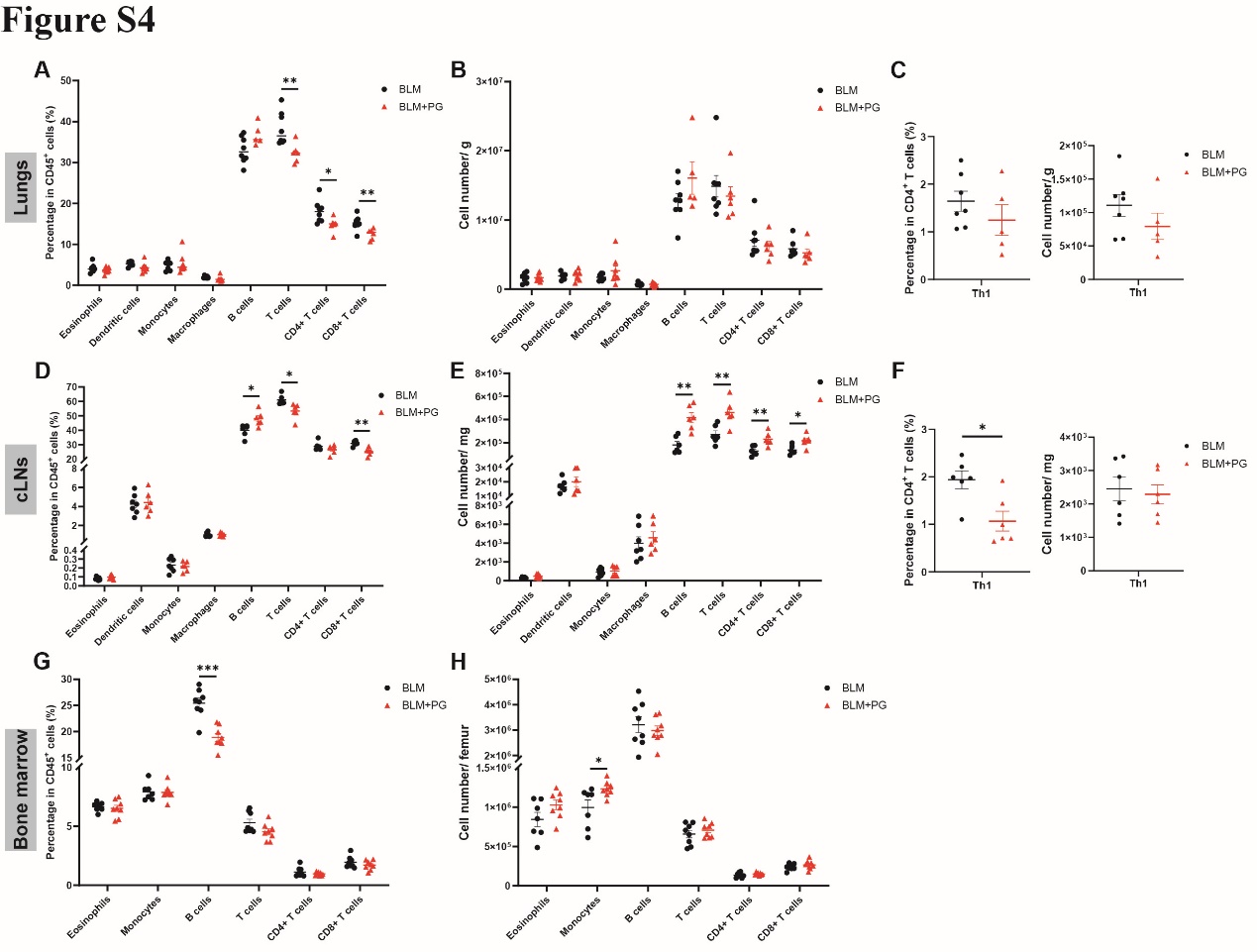


**Fig. S4 Effects of *Pg* on immune cells in lungs, cLNs and bone marrow of mice with pulmonary fibrosis.** (A-B) Quantifications of immune cells in mouse lungs as percentages of CD45^+^ cells (A) and numbers (B) at 7 days post bleomycin challenge. Periodontitis was induced in mice of the PG group by ligation of molars and oral inoculation of *Pg*. (C) Quantifications of Th1 cells in mouse lungs as percentages of CD4^+^ T cells and numbers at 7 days post bleomycin challenge. (D-E) Quantifications of immune cells in mouse cLNs as percentages of CD45^+^ cells (D) and numbers (E) at 7 days post bleomycin challenge. (F) Quantifications of Th1 cells in mouse cLNs as percentages of CD4^+^ T cells and numbers at 7 days post bleomycin challenge. (G-H) Quantifications of immune cells in mouse bone marrow as percentages of CD45^+^ cells (G) and numbers (H) at 7 days post bleomycin challenge. n=6-8. Data are presented as mean ± SEM. Student’s t-test was used for statistical analysis. *P < 0.05, **P < 0.01, and ***P < 0.001.

**Table S1: List of primers for qRT-PCR**

| **Gene** | **Forward** | **Reverse** |
| --- | --- | --- |
| *Ctgf* | GGGCCTCTTCTGCGATTTC | ATCCAGGCAAGTGCATTGGTA |
| *Timp1* | GCAACTCGGACCTGGTCATAA | CGGCCCGTGATGAGAAACT |
| *TGF-β1* | CTTCAATACGTCAGACATTCGGG | GTAACGCCAGGAATTGTTGCTA |
| *Col1a1* | AGAGCGGAGAGTACTGGATCG | TCGAACGGGAATCCATCGGT |
| *TNF-α* | CCCTCACACTCAGATCATCTTCT | GCTACGACGTGGGCTACAG |
| *18S* | GTAACCCGTTGAACCCCATT | CCATCCAATCGGTAGTAGCG |
